# Modeling spatially dependent count time series data using COM-poisson INGARCH Models

**DOI:** 10.1371/journal.pone.0350497

**Published:** 2026-10-06

**Authors:** Stephanie Colwell, Prabhakar Chalise, Byron Gajewski, Isuru Panduka Ratnayake, Dinesh Pal Mudaranthakam

**Affiliations:** 1 Department of Biostatistics & Data Science, University of Kansas Medical Center, Kansas City, Kansas, United States of America; 2 The University of Kansas Cancer Center, University of Kansas Medical Center, Kansas City, Kansas, United States of America; Jahangirnagar University, BANGLADESH

## Abstract

Count time series frequently exhibit serial dependence and either overdispersion or underdispersion relative to the Poisson equidispersion assumption. When such counts are observed across multiple locations, spatial dependence often arises, resulting in complex spatio-temporal dependency structures that are not adequately accommodated by standard integer-valued generalized autoregressive conditional heteroskedasticity (INGARCH) models. To address these challenges, this study proposes a spatial Conway–Maxwell–Poisson INGARCH (SP-COM-INGARCH) framework that jointly models temporal dependence, spatial interaction, and varying dispersion. The proposed framework incorporates the Conway-Maxwell-Poisson (COM-Poisson) distribution, which accommodates both overdispersion and underdispersion through an additional dispersion parameter, while a spatially weighted lag structure captures dependence across neighboring locations. We further introduce covariate-adjusted identity-link and softplus-link extensions. In particular, the softplus formulation allows covariates to have negative effects while ensuring positivity of the conditional COM-Poisson centering parameter. For the SP-COM-INGARCH (1,1) process sufficient conditions for stationarity and ergodicity are established, and expressions for the conditional first- and second-order moments are derived. The finite-sample performance of conditional maximum likelihood estimation is evaluated through simulations under several overdispersion and underdispersion scenarios. Parameter recovery is generally satisfactory, with accuracy tending to improve as the length of the time-series increases. We illustrate the framework using weekly influenza-like illness counts from Arkansas, Kansas, Missouri, and Oklahoma with absolute humidity as a time-varying covariate. Across six competing COM-Poisson and generalized Poisson models, the softplus SP-COM-INGARCHX model achieved the lowest joint out-of-sample RMSE, while the baseline SP-COM-INGARCH model achieved the lowest joint out-of-sample MAE, although the differences among several models were small. Overall, the proposed SP-COM-INGARCH framework offers a flexible extension for modeling spatiotemporal count data with covariate effects and flexible dispersion while providing competitive out-of-sample forecasting performance.

## 1. Introduction

Count time series record the number of events occurring over time and arise frequently in epidemiology, medicine, economics, and environmental science [1–7]. Accurately modeling such data is essential because estimates of temporal dynamics, prediction uncertainty, and future counts often inform surveillance, planning, and decision-making. Poisson integer-valued generalized autoregressive conditional heteroscedasticity (INGARCH) models provide a natural observation-driven framework by allowing the current conditional intensity to depend on previous observations and conditional intensities [8]. However, the standard Poisson INGARCH model assumes conditional equidispersion, whereby the conditional variance equals the conditional mean. This assumption is often violated in practice. Clustering, unobserved heterogeneity, epidemic outbreaks, and reporting variability can produce overdispersion, whereas more regular or constrained count-generating mechanisms can produce underdispersion. Failure to account for these dispersion patterns can result in inadequate representation of variability, inaccurate uncertainty estimates, and diminished model-fitting and forecasting performance. Consequently, there is a need for INGARCH models that preserve temporal dependence while accommodating both overdispersed and underdispersed counts.

A further challenge arises when count time series are observed across multiple geographic locations. Many natural phenomena, including influenza incidence, exhibit both temporal and spatial variations. Weekly influenza-like illness (ILI) counts are recorded for specific geographic units, but disease activity in neighboring regions may be associated through geographic proximity, population mobility, shared environmental conditions, and disease transmission dynamics [9–13]. As a result, count series observed across locations should not be assumed independent. Models that account for temporal dependence and dispersion but ignore spatial interaction may inadequately characterize regional transmission patterns and yield misleading inference, uncertainty assessments, or forecasts [14]. These considerations motivate the development of spatial count time-series models capable of jointly capturing temporal dependence, spatial interaction, flexible dispersion, and covariate effects.

The Conway–Maxwell–Poisson (COM-Poisson) distribution provides a flexible basis for modeling dispersion because its additional parameter allows overdispersed, equidispersed, and underdispersed counts to be modeled within a unified family [15, 16]. Zhu [17] embedded the COM-Poisson distribution in an INGARCH framework, providing a temporal framework for count series that depart from the Poisson equidispersion assumption. However, this formulation does not incorporate spatial interaction across locations. The COM-Poisson distribution is not universally preferable to alternative count distributions: the negative-binomial distribution is well suited to overdispersed data but does not ordinarily accommodate underdispersion, whereas the generalized Poisson distribution can represent departures from equidispersion with a different mean–variance relationship and parameter constraints [16, 17]. Consequently, the COM-Poisson distribution offers a useful alternative when the direction or degree of dispersion is uncertain, although its normalizing constant adds computational complexity.

Related developments have emerged in the spatial count modeling literature. Chen and Chen [18] proposed spatio-temporal hurdle and spatial zero-inflated generalized-Poisson models for weekly dengue counts, incorporating distance-based spatial weights and meteorological covariates. These models were designed to address spatial dependence, overdispersion, and excess zeros. This work demonstrates the value of combining spatial interaction, count dynamics, and environmental information, but its focus differs from a COM-Poisson framework intended to accommodate both overdispersion and underdispersion.

Several other spatial and network extensions of INGARCH and related count autoregressive models have been proposed. Jahn et al. [19] developed approximately linear spatio-temporal INGARCH models using softplus response functions, permitting negative dependence parameters while preserving positivity. Armillotta and Fokianos [20] proposed count network autoregressive models based on specified neighborhood structures, with stability and inference results for fixed and increasing network dimensions. Maletz et al. [21] developed parsimonious spatio-temporal count autoregressive models that incorporate neighborhood effects and covariates while controlling the number of parameters. Guo and Zhu [22, 23] introduced negative-binomial network autoregressive models for overdispersed multivariate counts, including softplus and community-based formulations. Chen et al. [24] developed an empirical Bayes spatial hurdle INGARCH model for spatial count time series with excess zeros. Collectively, these approaches address spatial or network dependence, nonlinear effects, overdispersion, excess zeros, and covariate adjustment. Comparatively limited attention has been given to integrating the COM-Poisson distribution into a spatial INGARCH framework that jointly accommodates temporal dependence, distance-based spatial interaction, covariates, and both overdispersion and underdispersion.

To address this gap, we propose a spatial COM-Poisson INGARCH (SP-COM-INGARCH) framework that integrates INGARCH temporal dynamics, distance-based spatially weighted lags, and flexible dispersion modeling. We further develop SP-COM-INGARCHX and softplus SP-COM-INGARCHX extensions to incorporate spatial-unit-specific and time-varying covariates. The identity-link formulation provides an approximately linear interpretation when the conditional COM-Poisson centering parameter remains positive, whereas the softplus formulation permits negative covariate effects while ensuring positivity [19]. Corresponding spatial generalized-Poisson models are fitted under the same temporal, spatial, and covariate structures to provide a benchmark. Thus, the principal contribution of this study is not the spatial structure or the COM-Poisson distribution in isolation, but their integration within a unified INGARCH framework for spatially dependent count time series.

The study makes four main contributions. First, it formulates the SP-COM-INGARCH, SP-COM-INGARCHX, and softplus SP-COM-INGARCHX models for spatially dependent count time series. Second, for the SP-COM-INGARCH (1,1) process, it establishes sufficient conditions for stationarity and ergodicity and derives expressions for the conditional first- and second-order moments. Third, finite-sample performance of conditional maximum likelihood estimation is evaluated through simulation studies under overdispersion and underdispersion scenarios, including scenarios with negative covariate effects. Finally, the proposed models and their generalized Poisson counterparts are applied to weekly ILI counts from Arkansas, Kansas, Missouri, and Oklahoma. Absolute humidity is incorporated as a time-varying exogenous covariate because low absolute humidity has been linked to influenza virus survival, transmission, and seasonal epidemic onset [25, 26]. Model performance is evaluated using in-sample goodness-of-fit and prediction measures and rolling-origin one-step-ahead forecasts over a 52-week out-of-sample period.

## 2. Methods

### 2.1. Proposed models

#### 2.1.1. Conway-maxwell-poisson distribution.

This section provides a brief overview of the Conway–Maxwell–Poisson (COM-Poisson) distribution, which is used later in the proposed modeling framework. The COM-Poisson distribution was originally proposed by Conway and Maxwell [15] as a model for a queuing system with state-dependent service rates. Shmueli et al. [16] later studied its probabilistic and statistical properties and demonstrated its usefulness for modeling count data with both overdispersion and underdispersion. Sellers et al. [27] reviewed the methods and applications of COM-Poisson models. Sellers and Shmueli [28] compared COM-Poisson regression with Poisson, negative-binomial, and restricted generalized-Poisson regression models, illustrating its flexibility for count regression. More recently, Hawa et al. [29] proposed a bootstrap Liu-type estimator for COM-Poisson regression to address multicollinearity among predictors. In this study, the COM-Poisson distribution allows the proposed spatial count time-series framework to accommodate both overdispersed and underdispersed scenarios. Therefore, the COM-Poisson distribution is considered as a flexible modeling choice rather than as a universally superior count distribution.

The COM-Poisson distribution is given by:


$$\begin{array}{c} P(Y=y)=\;\frac{\lambda^{y}}{\left(y!{)}^{\nu }Z(\lambda ,\nu \right)}\;,\;\;\;\;\;y\;=\;\text{0},\;\text{1},\;\text{2},\;\ldots .., \end{array}$$ (1)

where


$$\begin{array}{c} Z(\lambda ,\nu )=\sum_{r=\text{0}}^{\infty }\frac{\lambda^{r}}{(r!{)}^{\nu }}, \end{array}$$

is the normalizing constant, λ>0,and ν>0. The boundary case ν=0 is permitted only when 0<λ<1, in which case the COM-Poisson distribution reduces to a geometric distribution. Here, λ is a rate parameter and ν is the dispersion parameter. The distribution represents overdispersion when ν<1, equidispersion when ν=1, and underdispersion when ν>1. Thus, the COM-Poisson distribution can accommodate overdispersed, equidispersed, and underdispersed count data [16].

The COM-Poisson distribution includes several standard count distributions as special or limiting cases. When ν=1, the COM-Poisson distribution reduces to the Poisson distribution. When ν=0 and 0<λ<1, it reduces to the geometric distribution. For fixed λ, as ν→∞, the COM-Poisson distribution converges to the Bernoulli distribution with probability parameter λ/(1+λ) [16].

The COM-Poisson distribution is also a two-parameter exponential-family distribution [16]. For Y~COM-Poisson (λ,ν), higher-order moments can be expressed recursively. In particular, the *r*^th^ moment recursion is given by [16]:


$$\begin{array}{c} E\left(Y^{r+\text{1}}\right)=\left\{ \begin{array}{cc} \lambda \left[{E(Y+\text{1})}^{\text{1}-\nu }\right] & r=\text{0}\\ \lambda \frac{\partial }{\partial \lambda }E\left(Y^{r}\right)+E(Y)E\left(Y^{r}\right) & r>\text{0} \end{array}\right.\text{ .} \end{array}$$ (2)

The mean and variance of the COM-Poisson distribution can be written in terms of the normalizing constant Z(λ,ν)as [16, 30]


$$\begin{array}{c} \;E(Y)=\frac{\partial logZ(\lambda ,\nu )}{\partial log\lambda }=\lambda \frac{\partial logZ(\lambda ,\nu )}{\partial \lambda }, \end{array}$$ (3)

and


$$\begin{array}{c} Var(Y)=\frac{\partial E(Y)}{\partial log\lambda }=\lambda \frac{\partial E(Y)}{\partial \lambda }. \end{array}$$

Because the normalizing constant Z(λ,ν) generally has no closed-form expression, numerical or approximate methods are needed for likelihood evaluation and moment calculation. The asymptotic approximation proposed by Minka et al. [30] is given by:


$$\begin{array}{c} Z(\lambda ,\nu )=\frac{exp\left(\nu \lambda^{\frac{\text{1}}{\nu }}\right)}{{\left(\text{2}\pi \lambda^{\frac{\text{1}}{\nu }}\right)}^{\frac{\nu -\text{1}}{\text{2}}}\sqrt{\nu }}\left(\text{1}+O\left(\lambda^{\frac{-\text{1}}{\nu }}\right)\right). \end{array}$$ (4)

Using this approximation, the mean and variance of the COM-Poisson distribution can be approximated as


$$\begin{array}{c} E(Y)\approx \lambda^{\text{1}/\nu }-\frac{\nu -\text{1}}{\text{2}\nu }, \end{array}$$ (5)

and


$$\begin{array}{c} Var(Y)\approx \frac{\lambda^{\text{1}/\nu }}{\nu }.\; \end{array}$$

For fixed ν>0, these are large- λ asymptotic approximations. Their accuracy generally improves as λ increases and is lowest when both λ and ν are small [16, 30]. When the asymptotic approximation is insufficiently accurate, the normalizing constant and moments can instead be evaluated numerically [30].

An interpretative limitation of the original COM-Poisson formulation is that λ is not generally equal to the mean. Although λ corresponds to the mean in the special case ν=1, it can differ substantially from the mean when ν≠1. This can make interpretation difficult, especially in regression and time series modeling. Guikema and Coffelt [31] proposed a reparametrized COM-Poisson formulation that provides a more interpretable centering parameter. The reparametrized probability mass function is


$$\begin{array}{c} P(Y=y)=\frac{\text{1}}{S(\mu ,\nu )}{\left(\frac{\mu^{y}}{y!}\right)}^{\nu },\;y=\text{0},\text{1},\text{2},\ldots \; \end{array}$$ (6)

and


$$\begin{array}{c} S(\mu ,\nu )=\sum_{r=\text{0}}^{\infty }{\left(\frac{\mu^{r}}{r!}\right)}^{\nu }. \end{array}$$

Under this reparameterization, μ>0 and ν>0 are required. If ν=0, the substitution $\lambda =\mu^{\nu }$ gives λ = 1, for which the normalizing constant diverges. The parameter μ is related to the center of the distribution and is closely connected to the mode, making it more interpretable than λ in many applications. However, μ should not generally be interpreted as the exact mean when ν≠1 [31]. This reparameterization is obtained through the substitution $\mu =\lambda^{\text{1}/\nu }$, or equivalently, $\lambda =\mu^{\nu }$, allowing ν to retain its role as the dispersion or shape parameter. Under this parameterization, 0<ν<1 corresponding to overdispersion, ν=1 to equidispersion, and ν>1 to underdispersion [31]. The reparameterization provides a centering parameter to which an appropriate link function can be applied in regression and time-series models [31].

For the reparametrized COM-Poisson distribution, the exact mean of Y is


$$\begin{array}{c} E(Y)=\frac{{\sum_{r=\text{0}}^{\infty }r\left(\frac{\mu^{r}}{r!}\right)}^{\nu }}{S(\mu ,\nu )}.\; \end{array}$$ (7)

The second moment is $E\left(Y^{\text{2}}\right)=\frac{{\sum_{r=\text{0}}^{\infty }r^{\text{2}}\left(\frac{\mu^{r}}{r!}\right)}^{\nu }}{S(\mu ,\nu )}$. Therefore, the variance can be expressed as


$$\begin{array}{c} Var(Y)=\frac{\text{1}}{\nu }\frac{\partial E(Y)}{\partial \text{log}(\mu )}.\; \end{array}$$ (8)

These exact moment expressions follow from the original COM-Poisson formulas after applying the substitution $\lambda =\mu^{\nu }$ [16, 31]. The corresponding asymptotic approximations are


$$\begin{array}{c} E(Y)\approx \mu -\frac{\nu -\text{1}}{\text{2}\nu },\; \end{array}$$ (9)

and

$var(Y)\approx \frac{\mu }{\nu }.$

In the remainder of this paper, the reparametrized COM-Poisson distribution in Equation (6) is used. Accordingly, μ is interpreted as the conditional COM-Poisson intensity parameter or centering parameter rather than the exact conditional mean when ν≠1 [31]. This parameterization is used in the proposed INGARCH-type model because it improves interpretability while retaining the ability to model overdispersion and underdispersion.

In the next section, the reparametrized COM-Poisson distribution is embedded within the INGARCH framework to accommodate temporal dependence while retaining dispersion flexibility. Building on this temporal structure, the model is then extended to incorporate spatial dependence, allowing neighboring locations to interact through a spatially weighted lag structure. The resulting spatial Conway–Maxwell–Poisson INGARCH (SP-COM-INGARCH) formulation provides a unified framework for modeling spatially dependent count time series.

#### 2.1.2 Spatial COM-Poisson INGARCH Models.

The integer-valued generalized autoregressive conditional heteroscedasticity (INGARCH) framework provides the temporal foundation of the proposed model. It is an observation-driven approach in which a time-varying conditional mean or intensity depends on past observations and past conditional means or intensities [8]. A general INGARCH model of orders *p* and *q* (INGARCH (p,q)) can be written as:


$$\begin{array}{c} \eta_{t}=\omega +\sum_{r=\text{1}}^{p}\alpha_{r}Y_{t-r}+\sum_{m=\text{1}}^{q}\beta_{m}\eta_{t-m}, \end{array}$$

where, $\omega >\text{0};\alpha_{r}\geq \text{0},r=\text{1},\ldots ,p$, and $\beta_{m}\geq \text{0},m=\text{1},\ldots ,q$. In the conventional mean-parameterized formulation $\eta_{t}=E\left(Y_{t}|F_{t-\text{1}}\right)$. The parameter ω represents the baseline level of the conditional mean or intensity $\eta_{t}$. The coefficients $\alpha_{r}$ measure the effects of the previous observed counts, whereas the coefficients $\beta_{m}$ capture dependence through the previous conditional means or intensities. For the standard mean-parameterized INGARCH (p,q) process, a commonly used sufficient condition for stationarity is $\sum_{r=\text{1}}^{p}\alpha_{r}+\sum_{m=\text{1}}^{q}\beta_{m}<\text{1}$.

Based on the COM-Poisson INGARCH model of Zhu [17] the proposed spatial extension is defined as follows. Consider the observed count $Y_{i,t}$ observed at a spatial unit i and time t, where i=1,…,K and t=1,…,N. Here K is the number of spatial units and Nis the number of temporal observations. Let $F_{t}$ denote the σ− field generated by the observed history from all spatial units up to time t, that is, $F_{t}=\sigma \left\{Y_{i,r}:i=\text{1},\ldots ,K,r\leq t\right\}$. Conditional on the past information $F_{t-\text{1}}$, the observations $Y_{i,k},\ldots ,Y_{K,t}$ are assumed to be conditionally independent, with spatial dependence introduced through their conditional centering parameters. Specifically,


$$\begin{array}{c} Y_{i,t}|F_{t-\text{1}}\sim COM-Poisson\left(\mu_{i,t},\nu_{i}\right)\text{.} \end{array}$$

Here, $\mu_{i,t}>\text{0}$ denotes the conditional COM-Poisson centering parameter for spatial unit i at time t, and $\nu_{i}>\text{0}$ denotes the location-specific dispersion parameter. The parameter $\mu_{i,t}$ controls the center or level of the conditional distribution, but it is not generally equal to the conditional mean when $\nu_{i}\neq \text{1}.$ The conditional mean is determined jointly by $\mu_{i,t}$ and $\nu_{i}$,is given by: $E\left(Y_{i,t}|F_{t-\text{1}}\right)=m_{\nu_{i}}\left(\mu_{i,t}\right),$where the exact conditional mean function is $m\nu_{i}\left(\mu i,t\right)=\frac{\sum_{r=\text{0}}^{\infty }r{\left(\frac{\mu_{i,t}^{r}}{r!}\right)}^{\nu_{i}}}{S\left(\mu_{i,t},\nu_{i}\right)}$ and$S\left(\mu_{i,t}\nu_{i}\right)=\sum_{r=\text{0}}^{\infty }{\left(\frac{\mu_{i,t}^{r}}{r!}\right)}^{\nu_{i}}$.

In its general form, the SP-COM-INGARCH (p,q) model specifies the conditional centering parameter as


$$\begin{array}{c} \mu_{i,t}=\omega_{i}+\sum_{r=\text{1}}^{p}\alpha_{i,r}\sum_{j=\text{1}}^{K}{\gamma_{ji}Y}_{j,t-r}+\sum_{m=\text{1}}^{q}\beta_{i,m}\mu_{i,t-m}, \end{array}$$ (10)

where $\omega_{i}>\text{0},\alpha_{i,r}\geq \text{0},$ and $\beta_{i,m}\geq \text{0}$, and $\gamma_{ji}>\text{0}$ denotes the spatial weight from unit j to uniti as defined below. The order p determines the number of lagged spatial-count terms included in the model, whereas q determines the number of lagged conditional centering parameters.

Equation (10) demonstrates that the proposed framework is not restricted to first-order dependence. However, an SP-COM-INGARCH (p,q) model contains kp+kq location specific dynamic coefficients, in addition to its spatial and distributional parameters. Increasingp or q may therefore complicate estimation and weaken parameter identifiability. Therefore, the present study focuses on the parsimonious (1,1) specification.

Setting p=q=1, the SP-COM- INGARCH (1,1) model is


$$\begin{array}{c} \mu_{i,t}=\omega_{i}+\alpha_{i}\sum_{j=\text{1}}^{K}\gamma_{ji}Y_{j,t-\text{1}}+\beta_{i}\mu_{i,t-\text{1}}, \end{array}$$ (11)

where $\alpha_{i}=\alpha_{i,\text{1}}\geq \text{0},$ and $\beta_{i}=\beta_{i,\text{1}}\geq \text{0}$. The parameter $\omega_{i}$ represents the baseline level of the conditional centering parameter for spatial unit i. Because the spatial weights are normalized, $\alpha_{i}$ controls the overall magnitude of the lagged-count effect. The component with j=i represents feedback from the unit’s own lagged count, whereas the component with j≠i represent lagged cross-location effects. The parameter $\beta_{i}$ measures persistence through the previous conditional centering parameter. The equation (11) combined temporal persistence and spatial interaction within a single dynamic framework.

Rather than using a binary neighborhood matrix based only on shared spatial boundaries, this study follows the continuous distance-based weighting approach of Chen and Chen [18]. Let $s_{i}={\left(s_{i,\text{1}},s_{i,\text{2}}\right)}^{T}$, denote the geographical coordinates of the $i^{th}$ spatial unit, where i=1,…,K. The coordinates must be represented on a common spatial scale so that the distances are comparable. The spatial weight from unit j to unit i is


$$\begin{array}{c} \gamma_{ji}=\frac{d_{ji}}{\sum_{l=\text{1}}^{K}d_{li}}, \end{array}$$

where,


$$\begin{array}{c} d_{ji}=\text{exp}\left[-{\left(\frac{\left\|s_{j}-s_{i}\right\|}{\kappa }\right)}^{\tau }\right], \end{array}$$ (12)

and


$$\begin{array}{c} \left\|s_{j}-s_{i}\right\|=\sqrt{{\left(s_{j,\text{1}}-s_{i,\text{1}}\right)}^{\text{2}}+{\left(s_{j,\text{2}}-s_{i,\text{2}}\right)}^{\text{2}}}. \end{array}$$

For each receiving spatial unit i, the weights satisfy


$$\begin{array}{c} \sum_{j=\text{1}}^{K}\gamma_{ji}=\text{1}. \end{array}$$

Here, 0<τ<2 and κ>0 are spatial decay parameters, and $\left\|s_{j}-s_{i}\right\|$ denotes the Euclidean distance between spatial units i and j. The parameter κ controls the spatial range: smaller values concentrate the normalized weights on nearby units, whereas larger values assign relatively more weight to distant units. The parameter τ controls the shape of the distance-decay curve. Consequently, spatial influence decreases smoothly with distance rather than being restricted to binary adjacency relationships [18].

For clarity, the SP-COM-INGARCH model without exogenous covariates is referred to throughout this manuscript as the baseline SP-COM-INGARCH model, and the two terms are used interchangeably. This model serves as the reference specification for comparison with the identity-link and softplus SP-COM-INGARCHX extensions discussed in section 2.1.3.

#### 2.1.3 SP-COM-INGARCHX model with covariates and softplus extension.

The baseline SP-COM-INGARCH (1,1) model can be extended to incorporate spatial-unit-specific and time-varying covariates. Let $X_{i,t}\epsilon R^{d}$ denote the vector of d covariates observed for spatial unit i at time t, and define $X_{t}={\left(X_{\text{1},t}^{T},\ldots ,X_{K,t}^{T}\right)}^{T}$. For the covariate models, let $H_{t}$ denote the augmented information set containing the count history through time t−1 and the covariates observed through time t. Specifically, $H_{t}$ is defined as $H_{t}=\sigma \left({Y_{i,s}:i=\text{1},\ldots ,K,s\leq t-\text{1};X}_{s}:s\leq t\right)$. The resulting model, denoted the SP-COM-INGARCHX (1 1) model, is defined as

$Y_{i,t}|H_{t}\sim$COM-Poisson$\left(\mu_{i,t},\nu_{i}\right),$

with


$$\begin{array}{c} \;\mu_{i,t}=\omega_{i}+\alpha_{i}\sum_{j=\text{1}}^{K}\gamma_{ji}Y_{j,t-\text{1}}+\beta_{i}\mu_{i,t-\text{1}}+\phi_{i}^{T}X_{i,t}.\; \end{array}$$

Here, $X_{i,t}$ denotes the vector of covariates observed for spatial unit i at time t, and $\phi_{i}\epsilon R^{d}$denotes the corresponding vector of location-specific covariate coefficients. The covariate vector $X_{i,t}$ is assumed to be observed and available when specifying the conditional distribution of $Y_{i,t}$. If $X_{i,t}$ is not available at time t, it must be forecast or otherwise estimated using only the information available at that time, alternatively a lagged covariate vector may be used. The constraints on $\omega_{i},\alpha_{i}$, and $\beta_{i}$ remain as specified in Section 2.1.2. This extension allows the conditional COM-Poisson centering parameter $\mu_{i,t}$ to incorporate external spatial or temporal information in addition to spatially weighted lagged counts and the previous conditional centering parameter. More generally, the higher-order SP-COM-INGARCHX (p,q) model is obtained by adding covariate component to the general SP-COM-INGARCH (p,q) specification in Equation (10):


$$\begin{array}{c} \mu_{i,t}=\omega_{i}+\sum_{r=\text{1}}^{p}\alpha_{i,r}\sum_{j=\text{1}}^{K}{\gamma_{ji}Y}_{j,t-r}+\sum_{m=\text{1}}^{q}\beta_{i,m}\mu_{i,t-m}+\phi_{i}^{T}X_{i,t}\text{.} \end{array}$$ (14)

In the identity-link SP-COM-INGARCHX model in Equation (13), the conditional COM-Poisson centering parameter is specified directly by the linear predictor. Although this formulation is simple and interpretable, it may not guarantee positivity when covariates are included. Specifically, a negative covariate coefficient, or a sufficiently large covariate value associated with a negative coefficient, can produce a $\mu_{i,t}\leq \text{0}$. This is problematic because the COM-Poisson distribution requires $\mu_{i,t}>\text{0}$. Therefore, the identity-link formulation is valid only when the entire linear predictor is constrained to remain strictly positive at every time point.

A natural way to address this limitation is to replace the identity response function with a softplus response function. Softplus INGARCH models were introduced in the univariate INGARCH framework to preserve positivity while allowing approximately linear dependence structures [32]. Ratnayake and Samaranayake [33] extended the softplus INGARCH framework to incorporate exogenous variables in a zero-inflated INGARCHX model. The softplus formulation has also been applied to spatio-temporal INGARCH models, where it preserves positivity while retaining approximately linear dynamics when the linear predictor is sufficiently positive [19].

Motivated by these developments, a softplus version of the SP-COM-INGARCHX model is considered when negative covariate effects are permitted. For the general softplus SP-COM-INGARCHX (p,q) model, let $\psi_{i,t}$ denote the unconstrained linear predictor. The softplus model is defined by


$$\begin{array}{c} \psi_{i,t}=\omega_{i}+\sum_{r=\text{1}}^{p}\alpha_{i,r}\sum_{j=\text{1}}^{K}{\gamma_{ji}Y}_{j,t-r}+\sum_{m=\text{1}}^{q}\beta_{i,m}\mu_{i,t-m}+\phi_{i}^{T}X_{i,t}\; \end{array}$$ (15)

The conditional COM-Poisson centering parameter is then defined using the softplus response function:


$$\begin{array}{c} \mu_{i,t}={softplus}_{c}\left(\psi_{i,t}\right)=clog\left(\text{1}+\text{exp}\left(\frac{\psi_{i,t}}{c}\right)\right),\;c>\text{0.} \end{array}$$

Setting p=q=1 gives the softplus SP-COM-INGARCHX model evaluated in this study. The parameter c controls the scale and smoothness of the softplus response function. In the present formulation, c is treated as an additional positive scale parameter and is estimated jointly with the other model parameters.

Because $softplus_{c}(z)=clog\left(\text{1}+\text{exp}\left(\frac{z}{c}\right)\right)>\text{0},\forall z\in R,\;$ the softplus formulation guarantees that $\mu_{i,t}>\text{0}$, for every spatial unit i and time point t, even when some covariate coefficients are negative. Thus, the conditional COM-Poisson distribution remains well-defined. Moreover, because $softplus_{c}(z)\approx z$ when $\frac{z}{c}$ is sufficiently large and positive, the formulation retains an approximately linear interpretation while ensuring positivity [19, 32].

In this study, the identity-link SP-COM-INGARCHX model is used under the restriction that its linear predictor remains strictly positive. The softplus formulation is used as a positivity-preserving alternative when negative covariate effects are included or when positivity of the identity-link predictor cannot otherwise be guaranteed.

#### 2.1.4 Stationarity and Ergodicity.

This section establishes a sufficient contraction condition for the existence of a unique stationary and ergodic solution of the proposed SP-COM-INGARCH (1 1) process. Let $Y_{t}={\left(Y_{\text{1},t},\ldots ,Y_{K,t}\right)}^{T}$, denote the vector of counts across the K spatial units at time t, and let $\mu_{t}={\left(\mu_{\text{1},t},\ldots ,\mu_{K,t}\right)}^{T}$, denote the corresponding vector of conditional COM-Poisson centering parameters. Define $\omega ={\left(\omega_{\text{1}},\ldots ,\omega_{K}\right)}^{T}$, $A=diag\left(\alpha_{\text{1}},\ldots ,\alpha_{K}\right)$, $B=diag\left(\beta_{\text{1}},\ldots ,\beta_{K}\right)$, and let G denote the K×K spatial weight matrix with entries $G_{ij}=\gamma_{ji}$. The model in Equation (11) can be written as


$$\begin{array}{c} \mu_{t}=\omega +AGY_{t-1}+B\mu_{t-1}\text{.} \end{array}$$ (16)

Because the spatial weights are normalized according to Equation (12), $\gamma_{ji}>\text{0}$ and $\sum_{j=\text{1}}^{K}\gamma_{ji}=\text{1}$ for each spatial unit i.

For spatial unit i, define the exact conditional COM-Poisson mean function as


$$\begin{array}{c} m_{i}(u)=E\left(Y_{i.t}|\mu_{i,t}=u,\nu_{i}\right)\text{.} \end{array}$$

Because u is a COM-Poisson centering parameter rather than the exact conditional mean when $\nu_{i}\neq \text{1}$, the stability condition must account for the sensitivity of $m_{i}(u)$ to changes in u. For the reparametrized COM-Poisson distribution,


$$\begin{array}{c} m_{i}^{\prime }(u)=\frac{\nu_{i}}{u}var\left(Y_{i.t}|\mu_{i,t}=u,\nu_{i}\right)\text{.} \end{array}$$

As shown in Lemma S1 of S1 Appendix, $m_{i}$ is continuously differentiable, with the derivative given above. For the contraction result, assume that this derivative is bounded on the admissible state space, and define $\left[\omega_{i},\infty \right)$, and define


$$\begin{array}{c} L_{i}=\underset{u\geq \omega_{i}}{\text{sup}}\left|m_{i}^{\prime }(u)\right|=\underset{u\geq \omega_{i}}{\text{sup}}\left|\frac{\nu_{i}}{u}var\left(Y_{i.t}|\mu_{i,t}=u,\nu_{i}\right)\right|<\infty \text{.} \end{array}$$

Under this assumption, $m_{i}$ is Lipschitz continuous on $\left[\omega_{i},\infty \right)$ with Lipschitz constant $L_{i}$. Let $D_{L}=diag\left(L_{\text{1}},\ldots ,L_{k}\right)$. A sufficient contraction condition for the proposed SP-COM-INGARCH (1,1) process is


$$\begin{array}{c} \rho \left(AGD_{L}+B\right)<\text{1.} \end{array}$$ (17)

Here, ρ(.) denotes the spectral radius of a matrix. Under this condition, the recursion admits a unique causal and strictly stationary solution with finite first moments, and the joint process

${\left\{\left(Y_{t},\mu_{t}\right)\right\}}_{t\epsilon Z}$is ergodic. Because $AGD_{L}+B$, is a nonnegative matrix, a directly verifiable sufficient condition is obtained from its maximum row sum:


$$\begin{array}{c} \alpha_{i}\sum_{j=\text{1}}^{K}\gamma_{ji}L_{j}+\beta_{i}<\text{1},\;i=\text{1},\ldots ,K. \end{array}$$

Let


$$\begin{array}{c} L_{max}=\underset{\text{1}\leq j\leq K}{\text{max}}L_{j}\text{.} \end{array}$$

Because the spatial weights satisfy $\sum_{j=\text{1}}^{K}\gamma_{ji}=\text{1}$,the simpler but potentially more restrictive condition


$$\begin{array}{c} L_{max}\alpha_{i}+\beta_{i}<\text{1},i=\text{1},\ldots ,K, \end{array}$$

is also sufficient. In the Poisson special case, $\nu_{i}=\text{1}\forall i$, the conditional mean function is $m_{i}(u)=u$, and hence $L_{i}=\text{1}$. Condition (17) then becomes to ρ(AG+B)<1. The corresponding row-sum sufficient condition reduces to $\alpha_{i}+\beta_{i}<\text{1},,i=\text{1},\ldots ,K$, which is the familiar sufficient stability condition for a mean-parameterized INGARCH process.

For the reparametrized COM-Poisson model with $\nu_{i}\neq \text{1}$, $\mu_{i,t}$ is a centering parameter rather than the exact conditional mean. Therefore, the general Lipschitz-based contraction condition stated above is used instead of relying only on $\alpha_{i}+\beta_{i}<\text{1}$. The exact conditional first- and second-order moments of $Y_{i,t}|F_{t-\text{1}}$ are provided in Equations (7) and (8), with $\mu =\mu_{i,t}$and $\nu =\nu_{i}$. A formal proof of the stationarity and ergodicity result, including existence, uniqueness, and finite first moments, is provided in S1 Appendix.

#### 2.1.5 Maximum Likelihood Estimation.

The parameters of the proposed SP-COM-INGARCH (1,1) model are estimated by conditional maximum likelihood. Let


$$\begin{array}{c} \omega ={\left(\omega_{\text{1}},\ldots ,\omega_{K}\right)}^{T},\alpha ={\left(\alpha_{\text{1}},\ldots ,\alpha_{K}\right)}^{T},\beta ={\left(\beta_{\text{1}},\ldots ,\beta_{K}\right)}^{T},\text{\textasciitilde{}and\textasciitilde{}},\nu ={\left(\nu_{\text{1}},\ldots ,\nu_{K}\right)}^{T}\text{.} \end{array}$$

The full parameter vector is


$$\begin{array}{c} \theta ={\left(\omega^{T},\alpha^{T},\beta^{T},\nu^{T},\kappa ,\tau \right)}^{T}\text{.} \end{array}$$

For the SP-COM-INGARCHX models, θ is augmented by the covariate-coefficient vector $\phi ={\left(\phi_{\text{1}}^{T},\ldots ,\phi_{K}^{T}\right)}^{T}$. For the softplus model, θ is further augmented by the positive scale parameter c, which is estimated jointly with other model parameters.

Suppose the observed spatial count time series is


$$\begin{array}{c} \left\{Y_{i,t}:i=\text{1},\ldots ,K;t=\text{1},\ldots ,N\right\}\text{,} \end{array}$$

where K denotes the number of spatial units and N denotes the number of temporal observations. Conditional on the information set $F_{t-\text{1}}$ defined in Section 2.1.2, the observations $Y_{\text{1},t},\ldots ,Y_{K,t}$ are assumed to be conditionally independent across spatial units, with


$$\begin{array}{c} Y_{i,t}|F_{t-\text{1}}\sim COM-Poisson\left(\mu_{i,t},\nu_{i}\right)\text{,} \end{array}$$

where $\mu_{i,t}$ is given by the SP-COM-INGARCH (1 1) recursion in Equation (11). For the covariate models, $F_{t-\text{1}}$ is replaced by the augmented information set $H_{t}$ defined in Section 2.1.3. The conditional likelihood for the SP-COM-INGARCH model is therefore,


$$\begin{array}{c} L_{N}\left(\theta \right)=\prod_{t=\text{2}}^{N}\prod_{i=\text{1}}^{K}f\left(Y_{i,t}|F_{t-\text{1}};\theta \right)\text{,} \end{array}$$

where f(.) denotes the reparametrized COM-Poisson probability mass function defined in Equation (6). For the covariate models, the likelihood is conditioned on $H_{t}$ instead of $F_{t-\text{1}}.$ The likelihood is conditional on the initial values used to initialize the centering-parameter recursion. Therefore, the conditional log-likelihood contribution of the observation (i,t) is


$$\begin{array}{c} \;l_{i,t}\left(\theta \right)=\nu_{i}Y_{i,t}\text{log}\left(\mu_{i,t}\right)-\nu_{i}\text{log}\left(Y_{i,t}!\right)-\text{log}\left(S\left(\mu_{i,t},\nu_{i}\right)\right),\; \end{array}$$

where


$$\begin{array}{c} S\left(\mu_{i,t},\nu_{i}\right)={\sum_{r=\text{0}}^{\infty }\left(\frac{\mu_{i,t}^{r}}{r!}\right)}^{\nu_{i}}\text{.} \end{array}$$

The full conditional log-likelihood is therefore


$$\begin{array}{c} l_{N}\left(\theta \right)=\sum_{t=\text{2}}^{N}\sum_{i=\text{1}}^{K}l_{i,t}\left(\theta \right)\text{.} \end{array}$$

The conditional maximum likelihood estimator is defined as


$$\begin{array}{c} \hat{\theta }={argmax}_{\theta \in \Theta }l_{N}\left(\theta \right)\text{\textbackslash{}!,} \end{array}$$

where Θ denotes the admissible parameter space, satisfying the appropriate parameter restrictions and, where applicable, the sufficient stability conditions described in Section 2.1.4.

Because the COM-Poisson normalizing constant $S(\mu_{i,t},\nu_{i})$ does not have a closed-form expression, it is evaluated numerically using a finite truncation of the infinite series [27]. The parameter estimates are obtained by constrained numerical maximization of the conditional log-likelihood over the admissible parameter space Θ, incorporating the positivity restrictions and sufficient stability conditions described in Section 2.1.4. Because the normalizing constant must be evaluated repeatedly for every spatial unit and time point during the optimization, estimation of the SP-COM-INGARCH model is computationally more demanding than estimation of the corresponding spatial generalized Poisson model.

To express the score functions, let $\vartheta ={\left(\omega^{T},\alpha^{T},\beta^{T},\kappa ,\tau \right)}^{T}$ denote the parameter vector excluding the dispersion parameters. For the covariate models, ϑ is augmented by ϕ. For the softplus model, it is further augmented by c. The score function with respect to the dispersion parameter $\nu_{i}$is


$$\begin{array}{c} \;\frac{\partial l_{i,t}\left(\theta \right)}{\partial \nu_{i}}=Y_{i,t}\text{log}\left(\mu_{i,t}\right)-\text{log}\left(Y_{i,t}!\right)-\frac{\text{1}}{S\left(\mu_{i,t},\nu_{i}\right)}\sum_{r=\text{0}}^{\infty }\left(r\text{log}\mu_{i,t}-\text{log}(r!)\right){\left(\frac{\mu_{i,t}^{r}}{r!}\right)}^{\nu_{i}}\;. \end{array}$$

The score function with respect to ϑ is


$$\begin{array}{c} \;\frac{\partial l_{i,t}(\theta )}{\partial \vartheta }=\left[\frac{\nu_{i}Y_{i,t}}{\mu_{i,t}}-\frac{\text{1}}{S\left(\mu_{i,t},\nu_{i}\right)}\sum_{r=\text{0}}^{\infty }r\nu_{i}\frac{\mu_{i,t}^{r\nu_{i}-\text{1}}}{(r!{)}^{\nu_{i}}}\right]\frac{\partial \mu_{i,t}}{\partial \vartheta }.\; \end{array}$$

The derivatives of $\mu_{i,t}$ follow recursively from the SP-COM-INGARCH (1,1) model specification. For the spatial parameters κ and τ, the recursive derivatives also account for the dependence of the spatial weights $\gamma_{ji}$ on these parameters. For the softplus model, the recursive derivatives additionally apply the chain rule using


$$\begin{array}{c} \frac{\partial \mu_{i,t}}{\partial \psi_{i,t}}=\frac{\text{exp}\left(\frac{\psi_{i,t}}{c}\right)}{\text{1}+\text{exp}\left(\frac{\psi_{i,t}}{c}\right)}=\frac{\text{1}}{\text{1}+\text{exp}\left(-\frac{\psi_{i,t}}{c}\right)}, \end{array}$$

and


$$\begin{array}{c} \frac{\partial \mu_{i,t}}{\partial c}=\frac{\text{1}}{\text{1}+\text{exp}\left(-\frac{\psi_{i,t}}{c}\right)}\frac{\partial \psi_{i,t}}{\partial c}+\text{log}\left[\text{1}+\text{exp}\left(\frac{\psi_{i,t}}{c}\right)\right]-\frac{\psi_{i,t}}{c}.\frac{\text{1}}{\text{1}+\text{exp}\left(-\frac{\psi_{i,t}}{c}\right)} \end{array}$$

Approximate standard errors are computed using the robust sandwich covariance estimator [34]


$$\begin{array}{c} \hat{var}\left(\hat{\theta }\right)={\hat{H}}_{N}^{-1}{\hat{J}}_{N}{\hat{H}}_{N}^{-1}, \end{array}$$

where


$$\begin{array}{c} {\hat{J}}_{N}=\sum_{t=2}^{N}U_{t}\left(\hat{\theta }\right){U_{t}\left(\hat{\theta }\right)}^{T} \end{array}$$

is the empirical outer product of the score vectors,

$U_{t}\left(\theta \right)=\frac{\partial l_{t}\left(\theta \right)}{\partial \theta },\text{\textasciitilde{}}{𝐥}_{𝐭}\left(\theta \right)=\sum_{i=\text{1}}^{K}l_{i,t}\left(\theta \right)$

and


$$\begin{array}{c} {\hat{H}}_{N}=-\sum_{t=\text{2}}^{N}\frac{\partial^{\text{2}}l_{t}\left(\theta \right)}{\partial \theta \partial \theta^{\text{T}}}{|}_{\theta =\hat{\theta }}, \end{array}$$

is the observed negative Hessian matrix. The approximate standard errors are obtained as the square roots of the diagonal elements of $\hat{var}\left(\hat{\theta }\right)$.

#### 2.1.6 Forecasting and residual diagnostics.

After estimating the model parameters, one-step-ahead forecasts are obtained using the information available through the current forecasting origin. For the SP-COM-INGARCH (1 1) model, the predicted conditional COM-Poisson centering parameter for spatial unit iat time t+1 is


$$\begin{array}{c} {\hat{\mu }}_{i,t+\text{1}|t}={\hat{\omega }}_{i}+{\hat{\alpha }}_{i}\sum_{j=\text{1}}^{K}{\hat{\gamma }}_{ji}Y_{j,t}+{\hat{\beta }}_{i}{\hat{\mu }}_{i,t}\text{.} \end{array}$$

For the SP-COM-INGARCHX model, the one-step-ahead centering parameter additionally incorporates the covariate vector observed at time t+1, which is assumed to be known or available at the forecasting origin t:


$$\begin{array}{c} {\hat{\mu }}_{i,t+\text{1}|t}={\hat{\omega }}_{i}+{\hat{\alpha }}_{i}\sum_{j=\text{1}}^{K}{\hat{\gamma }}_{ji}Y_{j,t}+{\hat{\beta }}_{i}{\hat{\mu }}_{i,t}\text{.} \end{array}$$

If ${𝐗}_{i,t+\text{1}}$ is unavailable at forecasting origin t, it must be forecast or otherwise estimated using only the information available through time t. For the softplus SP-COM-INGARCHX model, the corresponding linear predictor is first calculated and then transformed using the softplus response function. Because ${\hat{\mu }}_{i,t+\text{1}}$ is a COM-Poisson centering parameter rather than the exact conditional mean when $\nu_{i}\neq \text{1}$, the corresponding one-step-ahead point forecast is ${\hat{Y}}_{i,t+\text{1}|t}=m_{{\hat{v}}_{i}}\left({\hat{\mu }}_{i,t+\text{1}|t}\right)$, where ${m_{\hat{\nu }}}_{i}(\mu )$ denotes the reparametrized COM-Poisson mean function defined in Equation (7). Corresponding 95% one-step-ahead prediction intervals are obtained from the 2.5th and 97.5th percentiles of the fitted conditional COM-Poisson distribution.

Out-of-sample forecasting performance is evaluated using a one-step-ahead rolling-origin procedure. The model is first estimated using the initial training sample, and a forecast is generated for the next observation. After that observation becomes available, it is added to the training sample, the model is re-estimated using the expanded sample, and the next one-step-ahead forecast is generated. This process is repeated throughout the holdout period so that each forecast is based only on information available at the corresponding forecasting origin.

To assess model adequacy, standardized Pearson residuals [3] are calculated as:


$$\begin{array}{c} \;e_{i,t}=\frac{Y_{i,t}-E(Y_{i,t}|F_{t-\text{1}};\hat{\theta })}{\sqrt{Var(Y_{i,t}|F_{t-\text{1}};\hat{\theta })}}.\; \end{array}$$

For the covariate models, $F_{t-\text{1}}$ is replaced by $H_{t}$. Here, the fitted conditional mean and the fitted conditional variance are expressed as:


$$\begin{array}{c} E\left(Y_{i,t}|F_{t-\text{1}};\hat{\theta }\right)=\frac{\sum_{r=\text{0}}^{\infty }r{\left(\frac{{\hat{\mu }}_{i,t}^{r}}{r!}\right)}^{{\hat{\nu }}_{i}}}{S\left({\hat{\mu }}_{i,t},{\hat{\nu }}_{i}\right)}, \end{array}$$

$$\begin{array}{c} Var\left(Y_{i,t}|F_{t-\text{1}};\hat{\theta }\right)=\frac{\sum_{r=\text{0}}^{\infty }r^{\text{2}}{\left(\frac{{\hat{\mu }}_{i,t}^{r}}{r!}\right)}^{{\hat{\nu }}_{i}}}{S\left({\hat{\mu }}_{i,t}{\hat{\nu }}_{i}\right)}-{\left(E\left(Y_{i,t}|F_{t-\text{1}};\hat{\theta }\right)\right)}^{\text{2}}\text{.} \end{array}$$

where,


$$\begin{array}{c} S\left({\hat{\mu }}_{i,t}{\hat{\nu }}_{i}\right)=\sum_{r=\text{0}}^{\infty }{\left(\frac{{\hat{\mu }}_{i,t}^{r}}{r!}\right)}^{{\hat{\nu }}_{i}}\text{.} \end{array}$$

### 2.2. Data sources

Weekly counts of outpatient visits for influenza-like illness (ILI) were obtained for four U.S. states: Arkansas, Kansas, Missouri, and Oklahoma from the U.S. Outpatient Influenza-like Illness Surveillance Network (ILINet), available through the Centers for Disease Control and Prevention (CDC) FluView system [35]. The weekly ILI count reported in the ILITOTAL field was used as the outcome, and all observations were indexed by Morbidity and Mortality Weekly Report (MMWR) epidemiological year and week. The analytic series contained 751 consecutive epidemiological weeks, from MMWR week 40 of 2010 through week 8 of 2025, corresponding approximately to October 2010 through February 2025. For model development, the first 699 weeks (MMWR week 40 of 2010 through MMWR week 8 of 2024), were used as the in-sample estimation period. The subsequent 52 weeks (MMWR week 9 of 2024 through MMWR week 8 of 2025), were reserved for out-of-sample forecasting. The latitude and longitude coordinates of the 2020 state mean centers of population, obtained from the U.S. Census Bureau [36], were used only to calculate the pairwise interstate distances required to construct the spatial weight matrix.

A point-based estimate of absolute humidity was used as the state-specific exogenous covariate. Daily mean 2-m air temperature and relative humidity were obtained from the NASA POWER database for one prespecified location in each of the four states [37]. Daily absolute humidity (g/m^3^) was calculated using Bolton’s saturation vapor pressure approximation [38]. Each daily value was assigned to its corresponding MMWR epidemiological week, and the daily values were averaged within each week to obtain the weekly absolute humidity covariate.

All sources were publicly available and contained only aggregated surveillance or environmental data. The authors had no access to information that could identify individual participants during or after data collection.

## 3. Simulation study

A Monte Carlo simulation study was conducted to evaluate the finite-sample performance of the conditional maximum likelihood estimators for the proposed spatial COM-Poisson models. Three model families were examined: the SP-COM-INGARCH (1,1) model, the identity-link SP-COM-INGARCHX (1,1) model with positive covariate effects, and the softplus SP-COM-INGARCHX (1,1) model with negative covariate effects. Both overdispersed and underdispersed data-generating processes were considered for each model family.

All simulation settings involved K=4 spatial units arranged on a fixed 2×2 grid with coordinates (1,1),(1,2),(2,1), and (2,2). Euclidean distances between the spatial units were used to construct the spatial weights. The simulation scenarios covered different baseline count levels, degrees of temporal persistence, spatial dependence, covariate effects, and dispersion levels. For the identity-link SP-COM-INGARCHX (1,1) model, the parameter and covariate values were selected so that the COM-Poisson centering parameter ($\mu_{i,t}$) remained positive. In contrast, the softplus SP-COM-INGARCHX (1,1) model was evaluated under negative covariate effects to assess its ability to accommodate such effects while preserving positivity of the $\mu_{i,t}$.

For each simulation setting and each time series length, R=1000 independent datasets were generated for time-series lengths N=500 and N=1000. The process was initialized by setting $\mu_{i,\text{1}}=\omega_{i},i=\text{1},\ldots ,K$, after which the initial counts were generated from the corresponding COM-Poisson distributions. The values of $\mu_{i,t}$ and the counts were then generated recursively until a complete time series of length N was obtained. Conditional on the past and the covariates, where applicable, counts were generated independently across spatial units from the reparametrized COM-Poisson distribution using the *COMPoissonReg* package in R [39]. At each time point, including initialization, the model recursion was first used to obtain the conditional COM-Poisson centering parameter $\mu_{i,t}$. Because the *rcmp* [39] function uses the conventional rate parameter, the following conversion was applied:$\lambda_{i,t}=\mu_{i,t}^{\nu_{i}}$.

All simulations were performed in R using the *COMPoissonReg* [39] and *nloptr* [40] packages. For each generated dataset, the conditional log-likelihood was optimized using the derivative-free Constrained Optimization BY Linear Approximations *(COBYLA)* algorithm implemented in *nloptr* [40, 41]. The maximum number of objective-function evaluations was set to 5000, and the relative parameter and objective-function tolerances were both set to ${\text{1}\text{0}}^{-\text{6}}$. Estimation was performed subject to the admissible parameter bounds and persistence restrictions specified in the simulation programs.

For each replication, the complete and finite parameter estimates returned by the optimization procedure were recorded. Let $\theta_{h}$denote the true value of the h^th^ parameter and let ${\hat{\theta }}_{h}^{\left(r\right)}$denote its estimate from the r^th^ replication, where r=1,…,R. The mean estimate of the hth parameter was calculated as


$$\begin{array}{c} {\overset{―}{\hat{\theta }}}_{h}=\frac{\text{1}}{R}\sum_{r=\text{1}}^{R}{\hat{\theta }}_{h}^{\left(r\right)},\;R=\text{1}\text{0}\text{0}\text{0}\text{.} \end{array}$$

The empirical bias was calculated as


$$\begin{array}{c} Bias\left({\hat{\theta }}_{h}\right)={\overset{―}{\hat{\theta }}}_{h}-\theta_{h}, \end{array}$$

and mean absolute error (MAE) were calculated as


$$\begin{array}{c} MAE\left({\hat{\theta }}_{h}\right)=\frac{\text{1}}{R}\sum_{r=\text{1}}^{R}\left|{\hat{\theta }}_{h}^{\left(r\right)}-\theta_{h}\right|\text{.} \end{array}$$

The simulation results for the SP-COM-INGARCH, identity-link SP-COM-INGARCHX, and softplus SP-COM-INGARCHX models with order (1 1) are presented in the following subsections.

### 3.1. Simulation Study for the SP-COM-INGARCH (1 1) model

Six simulation scenarios were used to assess the estimation performance of the SP-COM-INGARCH (1 1) model under overdispersion and underdispersion. Three scenarios were examined under each dispersion setting. The scenarios differed in their baseline parameters $\left(\omega_{i}\right)$, overall dynamic persistence $\left(\alpha_{i}+\beta_{i}\right)$, and spatial parameters τ and κ. In the spatial-weight function, κ controls the spatial range of dependence, whereas τ controls the shape and rate of distance decay. The results for overdispersion $\left(\nu_{i}<\text{1}\right)$and underdispersion $\left(\nu_{i}>\text{1}\right)$ are presented in Sections 3.1.1 and 3.1.2, respectively.

#### 3.1.1 SP-COM-INGARCH (1,1) Overdispersion Scenarios.

The three overdispersion scenarios represented different baseline levels and dependence structures, with the dispersion parameters $\nu_{i}$ ranging from 0.45 to 0.70. Scenario O1 used small baseline parameters, low dynamic persistence, and spatial parameters τ=0.75, κ=0.30. Scenario O2 used moderate baseline parameters and moderate persistence, with τ=1.50, κ=0.50, whereas O3 used much larger baseline parameters, moderate persistence, and τ=1.20, κ=0.80. Table 1, Table 2, Table 3 present the true parameter values, mean estimates, empirical biases, and MAEs for scenarios O1–O3 at N=500and N=1000.

**Table 1:** SP-COM-INGARCH (1,1) simulation results under overdispersion: Scenario O1.

Parameter	Truth	Time Series Length N = 500			Time Series Length N = 1000		
		Mean Estimate	Bias	MAE	Mean Estimate	Bias	MAE
$\omega_{\text{1}}$	1.0000	1.1532	0.1532	0.2342	1.1614	0.1614	0.2040
$\omega_{\text{2}}$	1.3000	1.4922	0.1922	0.2843	1.4983	0.1983	0.2547
$\omega_{\text{3}}$	1.1000	1.3878	0.2878	0.3307	1.3968	0.2968	0.3138
$\omega_{\text{4}}$	1.5000	1.6161	0.1161	0.2746	1.6164	0.1164	0.2308
$\alpha_{\text{1}}$	0.1100	0.1087	−0.0013	0.0379	0.1078	−0.0022	0.0265
$\alpha_{\text{2}}$	0.0900	0.0892	−0.0008	0.0377	0.0892	−0.0008	0.0266
$\alpha_{\text{3}}$	0.1000	0.1062	0.0062	0.0388	0.1026	0.0026	0.0286
$\alpha_{\text{4}}$	0.0800	0.0774	−0.0026	0.0355	0.0779	−0.0021	0.0280
$\beta_{\text{1}}$	0.1900	0.2140	0.0240	0.1054	0.2140	0.0240	0.0837
$\beta_{\text{2}}$	0.2100	0.2219	0.0119	0.1028	0.2215	0.0115	0.0808
$\beta_{\text{3}}$	0.1800	0.1938	0.0138	0.0888	0.1969	0.0169	0.0715
$\beta_{\text{4}}$	0.2200	0.2250	0.0050	0.1083	0.2242	0.0042	0.0872
τ	0.7500	0.8179	0.0679	0.2980	0.8121	0.0621	0.2220
κ	0.3000	0.3529	0.0529	0.1146	0.3410	0.0410	0.1046
$\nu_{\text{1}}$	0.6500	0.9231	0.2731	0.2731	0.9241	0.2741	0.2741
$\nu_{\text{2}}$	0.5500	0.7524	0.2024	0.2024	0.7486	0.1986	0.1986
$\nu_{\text{3}}$	0.4500	0.6750	0.2250	0.2250	0.6731	0.2231	0.2231
$\nu_{\text{4}}$	0.7000	0.8730	0.1730	0.1730	0.8708	0.1708	0.1708

**Table 2:** SP-COM-INGARCH (1 1) simulation results under overdispersion: Scenario O2.

Parameter	Truth	Time Series Length N = 500			Time Series Length N = 1000		
		Mean Estimate	Bias	MAE	Mean Estimate	Bias	MAE
$\omega_{\text{1}}$	10.0000	10.1251	0.1251	1.2178	10.1489	0.1489	0.8890
$\omega_{\text{2}}$	15.0000	15.2243	0.2243	1.7937	15.1861	0.1861	1.3067
$\omega_{\text{3}}$	12.0000	12.1531	0.1531	1.5077	12.2061	0.2061	1.0828
$\omega_{\text{4}}$	17.0000	17.2890	0.2890	1.9206	17.1918	0.1918	1.4505
$\alpha_{\text{1}}$	0.2900	0.2857	−0.0043	0.0357	0.2891	−0.0009	0.0245
$\alpha_{\text{2}}$	0.3400	0.3337	−0.0063	0.0358	0.3381	−0.0019	0.0261
$\alpha_{\text{3}}$	0.2700	0.2655	−0.0045	0.0343	0.2683	−0.0017	0.0251
$\alpha_{\text{4}}$	0.3100	0.3038	−0.0062	0.0356	0.3078	−0.0022	0.0263
$\beta_{\text{1}}$	0.3100	0.3057	−0.0043	0.0527	0.3053	−0.0047	0.0362
$\beta_{\text{2}}$	0.2600	0.2569	−0.0031	0.0527	0.2573	−0.0027	0.0377
$\beta_{\text{3}}$	0.3300	0.3268	−0.0032	0.0534	0.3249	−0.0051	0.0355
$\beta_{\text{4}}$	0.2900	0.2861	−0.0039	0.0528	0.2875	−0.0025	0.0400
τ	1.5000	1.5253	0.0253	0.0677	1.5171	0.0171	0.0557
κ	0.5000	0.5004	0.0004	0.0692	0.4956	−0.0044	0.0494
$\nu_{\text{1}}$	0.6000	0.6145	0.0145	0.0333	0.6076	0.0076	0.0225
$\nu_{\text{2}}$	0.5000	0.5082	0.0082	0.0260	0.5059	0.0059	0.0185
$\nu_{\text{3}}$	0.4500	0.4582	0.0082	0.0244	0.4562	0.0062	0.0173
$\nu_{\text{4}}$	0.6000	0.6117	0.0117	0.0322	0.6064	0.0064	0.0231

**Table 3:** SP-COM-INGARCH (1 1) Simulation results under overdispersion: Scenario O3.

Parameter	Truth	Time Series Length N = 500			Time Series Length N = 1000		
		Mean Estimate	Bias	MAE	Mean Estimate	Bias	MAE
$\omega_{\text{1}}$	1000.0000	998.7940	−1.2060	17.5253	999.1782	−0.8218	23.3100
$\omega_{\text{2}}$	1500.0000	1507.8902	7.8902	24.6996	1514.6591	14.6591	33.3846
$\omega_{\text{3}}$	1200.0000	1211.9217	11.9217	23.8922	1215.8625	15.8625	30.0334
$\omega_{\text{4}}$	1700.0000	1700.9000	0.9000	24.5552	1703.0059	3.0059	30.5975
$\alpha_{\text{1}}$	0.2800	0.2810	0.0010	0.0047	0.2811	0.0011	0.0045
$\alpha_{\text{2}}$	0.3000	0.3001	0.0001	0.0053	0.2997	−0.0003	0.0059
$\alpha_{\text{3}}$	0.3200	0.3218	0.0018	0.0057	0.3217	0.0017	0.0062
$\alpha_{\text{4}}$	0.2900	0.2914	0.0014	0.0054	0.2914	0.0014	0.0053
$\beta_{\text{1}}$	0.3200	0.3216	0.0016	0.0076	0.3220	0.0020	0.0072
$\beta_{\text{2}}$	0.3000	0.3009	0.0009	0.0067	0.3006	0.0006	0.0069
$\beta_{\text{3}}$	0.2800	0.2778	−0.0022	0.0075	0.2778	−0.0022	0.0078
$\beta_{\text{4}}$	0.3100	0.3101	0.0001	0.0065	0.3101	0.0001	0.0066
τ	1.2000	1.2006	0.0006	0.0403	1.2029	0.0029	0.0435
κ	0.8000	0.7932	−0.0068	0.0148	0.7923	−0.0077	0.0152
$\nu_{\text{1}}$	0.7500	0.7322	−0.0178	0.0234	0.7306	−0.0194	0.0238
$\nu_{\text{2}}$	0.7000	0.6960	−0.0040	0.0152	0.6973	−0.0027	0.0153
$\nu_{\text{3}}$	0.6000	0.5862	−0.0138	0.0183	0.5861	−0.0139	0.0187
$\nu_{\text{4}}$	0.7000	0.6820	−0.0180	0.0232	0.6799	−0.0201	0.0237

As shown in Table 1, Scenario O1, characterized by small baseline values and low dynamic persistence, produced noticeable biases and MAEs for the baseline and spatial parameters. As the time-series length increased from N=500 to N=1000, most mean estimates moved closer to their true values, with corresponding reductions in absolute bias and MAE. Table 2 shows improved estimation under Scenario O2, with mean estimates generally close to their true values and relatively small biases and MAEs. In Scenario O3 (Table 3), the temporal, spatial, and dispersion parameters were also estimated accurately. Although the absolute MAEs of ${\hat{\omega }}_{i}$ were larger in O3, they were small compared with the substantially larger true baseline values.

Comparisons across Tables 1–3 indicate that estimation performance was generally better under the parameter combinations considered in O2 and O3 than under the low-baseline setting in O1. Increasing the time-series length generally reduced the absolute biases for most parameters and reduced the MAEs in O1 and O2. However, the improvement was less uniform for the large baseline parameters in O3. Overall, the conditional maximum likelihood estimator demonstrated satisfactory finite-sample performance across the overdispersion scenarios for the SP-COM-INGARCH (1 1) model, with estimation accuracy generally improving as the time-series length increased.

#### 3.1.2 SP-COM-INGARCH (1 1) Underdispersion Scenarios.

Three underdispersion scenarios were considered, with $\nu_{i}>\text{1}$ and values ranging from 1.20 to 1.80. Scenario U1 used small baseline parameters, low dynamic persistence, and spatial parameters τ=0.75 and κ=0.30. Scenario U2 combined moderate baseline parameters and moderate persistence with τ=1.50 and κ=0.50, whereas Scenario U3 used substantially larger baseline parameters, moderate persistence, and spatial parameters τ=1.20 and κ=0.80. Table 4, Table 5, Table 6 present the true parameter values, means estimates, empirical biases, and MAEs for U1-U3 at N=500 to N=1000.

**Table 4:** SP-COM-INGARCH (1,1) simulation results under underdispersion: Scenario U1.

Parameter	Truth	Time Series Length N = 500			Time Series Length N = 1000		
		Mean Estimate	Bias	MAE	Mean Estimate	Bias	MAE
$\omega_{\text{1}}$	1.0000	1.0380	0.0380	0.1167	1.0301	0.0301	0.0863
$\omega_{\text{2}}$	1.4000	1.4064	0.0064	0.1571	1.4084	0.0084	0.1067
$\omega_{\text{3}}$	1.2000	1.2026	0.0026	0.1523	1.2081	0.0081	0.0947
$\omega_{\text{4}}$	1.6000	1.6115	0.0115	0.1486	1.6154	0.0154	0.1032
$\alpha_{\text{1}}$	0.1200	0.1177	−0.0023	0.0376	0.1197	−0.0003	0.0281
$\alpha_{\text{2}}$	0.1000	0.0991	−0.0009	0.0385	0.1006	0.0006	0.0258
$\alpha_{\text{3}}$	0.0800	0.0795	−0.0005	0.0345	0.0801	0.0001	0.0253
$\alpha_{\text{4}}$	0.1100	0.1078	−0.0022	0.0385	0.1109	0.0009	0.0278
$\beta_{\text{1}}$	0.1800	0.1847	0.0047	0.0630	0.1954	0.0154	0.0477
$\beta_{\text{2}}$	0.2000	0.2026	0.0026	0.0731	0.2030	0.0030	0.0474
$\beta_{\text{3}}$	0.2200	0.2224	0.0024	0.0813	0.2205	0.0005	0.0501
$\beta_{\text{4}}$	0.1700	0.1723	0.0023	0.0531	0.1711	0.0011	0.0364
τ	0.7500	0.9140	0.1640	0.3385	0.9505	0.2005	0.3104
κ	0.3000	0.3444	0.0444	0.0896	0.3311	0.0311	0.0701
$\nu_{\text{1}}$	1.3000	1.6472	0.3472	0.3472	1.6475	0.3475	0.3475
$\nu_{\text{2}}$	1.5000	1.7102	0.2102	0.2120	1.7013	0.2013	0.2015
$\nu_{\text{3}}$	1.7000	1.9729	0.2729	0.2745	1.9608	0.2608	0.2608
$\nu_{\text{4}}$	1.4000	1.5705	0.1705	0.1738	1.5662	0.1662	0.1667

**Table 5:** SP-COM-INGARCH (1 1) simulation results under underdispersion: Scenario U2.

Parameter	Truth	Time Series Length N = 500			Time Series Length N = 1000		
		Mean Estimate	Bias	MAE	Mean Estimate	Bias	MAE
$\omega_{\text{1}}$	10.0000	10.0451	0.0451	0.8592	10.0630	0.0630	0.5992
$\omega_{\text{2}}$	15.0000	15.1116	0.1116	1.1281	15.0761	0.0761	0.8558
$\omega_{\text{3}}$	12.0000	12.0289	0.0289	0.9261	12.0619	0.0619	0.6547
$\omega_{\text{4}}$	17.0000	17.1803	0.1803	1.3794	17.0437	0.0437	0.9108
$\alpha_{\text{1}}$	0.3200	0.3168	−0.0032	0.0293	0.3173	−0.0027	0.0211
$\alpha_{\text{2}}$	0.2800	0.2754	−0.0046	0.0300	0.2780	−0.0020	0.0212
$\alpha_{\text{3}}$	0.3500	0.3470	−0.0030	0.0284	0.3482	−0.0018	0.0197
$\alpha_{\text{4}}$	0.3000	0.2958	−0.0042	0.0296	0.2986	−0.0014	0.0203
$\beta_{\text{1}}$	0.2800	0.2765	−0.0035	0.0287	0.2783	−0.0017	0.0197
$\beta_{\text{2}}$	0.3300	0.3285	−0.0015	0.0295	0.3287	−0.0013	0.0204
$\beta_{\text{3}}$	0.2500	0.2474	−0.0026	0.0249	0.2480	−0.0020	0.0163
$\beta_{\text{4}}$	0.3100	0.3071	−0.0029	0.0313	0.3092	−0.0008	0.0198
τ	1.5000	1.5040	0.0040	0.0463	1.5011	0.0011	0.0376
κ	0.5000	0.5060	0.0060	0.0467	0.5014	0.0014	0.0328
$\nu_{\text{1}}$	1.4000	1.4167	0.0167	0.0614	1.4084	0.0084	0.0395
$\nu_{\text{2}}$	1.6000	1.6205	0.0205	0.0691	1.6098	0.0098	0.0501
$\nu_{\text{3}}$	1.8000	1.8228	0.0228	0.0879	1.8149	0.0149	0.0582
$\nu_{\text{4}}$	1.5000	1.5200	0.0200	0.0687	1.5091	0.0091	0.0453

**Table 6:** SP-COM-INGARCH (1 1) simulation results under underdispersion: Scenario U3.

Parameter	Truth	Time Series Length N = 500			Time Series Length N = 1000		
		Mean Estimate	Bias	MAE	Mean Estimate	Bias	MAE
$\omega_{\text{1}}$	1000.0000	998.6428	−1.3572	16.9299	998.8170	−1.1830	23.6452
$\omega_{\text{2}}$	1500.0000	1508.4385	8.4385	22.6015	1520.3903	20.3903	36.8566
$\omega_{\text{3}}$	1200.0000	1208.6602	8.6602	18.9072	1218.1568	18.1568	29.6382
$\omega_{\text{4}}$	1700.0000	1699.4349	−0.5651	22.0176	1702.4970	2.4970	34.9402
$\alpha_{\text{1}}$	0.2700	0.2715	0.0015	0.0048	0.2713	0.0013	0.0046
$\alpha_{\text{2}}$	0.3100	0.3100	0.0000	0.0050	0.3094	−0.0006	0.0053
$\alpha_{\text{3}}$	0.2900	0.2923	0.0023	0.0053	0.2915	0.0015	0.0055
$\alpha_{\text{4}}$	0.3300	0.3312	0.0012	0.0057	0.3314	0.0014	0.0061
$\beta_{\text{1}}$	0.3300	0.3308	0.0008	0.0073	0.3319	0.0019	0.0074
$\beta_{\text{2}}$	0.2900	0.2911	0.0011	0.0065	0.2904	0.0004	0.0071
$\beta_{\text{3}}$	0.3200	0.3175	−0.0025	0.0072	0.3176	−0.0024	0.0075
$\beta_{\text{4}}$	0.2700	0.2708	0.0008	0.0062	0.2707	0.0007	0.0063
τ	1.2000	1.2037	0.0037	0.0410	1.1971	−0.0029	0.0355
κ	0.8000	0.7937	−0.0063	0.0144	0.7907	−0.0093	0.0159
$\nu_{\text{1}}$	1.2000	1.1892	−0.0108	0.0241	1.1848	−0.0152	0.0220
$\nu_{\text{2}}$	1.2500	1.2491	−0.0009	0.0241	1.2463	−0.0037	0.0223
$\nu_{\text{3}}$	1.3000	1.2897	−0.0103	0.0262	1.2890	−0.0110	0.0255
$\nu_{\text{4}}$	1.2000	1.1910	−0.0090	0.0229	1.1888	−0.0112	0.0220

As shown in Table 4, Scenario U1 yield mean estimates generally close to their true values for the baseline parameters and temporal parameters. However, τ and some dispersion parameters $\nu_{i}$ exhibited noticeable positive biases. Increasing the time series length form N=500 to N=1000 reduced the MAEs for the most parameters, although the biases of some spatial and dispersion parameters did not decrease uniformly. Scenario U2 (Table 5) showed the strongest overall estimation performance, with small biases and MAEs that decreased consistently as the time-series length increased. In Scenario U3 (Table 6), the temporal, spatial, and dispersion parameters were also estimated with good accuracy. The absolute MAEs of ${\hat{\omega }}_{i}$ were larger because the true baseline values were substantially larger, and improvements at N=1000 were less consistent for these parameters.

Across Tables 4–6, the temporal parameters $\alpha_{i}$ and $\beta_{i}$ were generally estimated accurately, although some spatial and dispersion parameters in U1 showed comparatively larger estimation errors. A longer time series produced the clearest improvement in U1 and U2, while the results for U3 remained comparatively stable except for the baseline parameters. Compared with the corresponding overdispersion scenarios in Tables 1–3, estimation performance was generally better under underdispersion, with smaller biases and MAEs for many parameters, although this advantage was not uniform. Overall, the conditional maximum likelihood estimator demonstrated satisfactory finite-sample performance for the SP-COM-INGARCH (1 1) model under underdispersion.

### 3.2. Simulation Study for the SP-COM-INGARCHX (1 1) model

Six scenarios were considered to evaluate the finite-sample performance of the conditional maximum likelihood estimator for the identity-link SP-COM-INGARCHX (1,1) model. The scenarios varied in the baseline parameters ($\omega_{i}$), dynamic persistence ($\alpha_{i}+\beta_{i}$), spatial parameters (τ and κ), dispersion parameters ($\nu_{i}$), and covariate effects ($\phi_{i}$). Only positive covariate effects were considered in this simulation study. Results for the overdispersion and underdispersion scenarios are presented in Sections 3.2.1 and 3.2.2, respectively.

#### 3.2.1 SP-COM-INGARCHX (1 1) Overdispersion Scenarios.

Three overdispersion scenarios were examined, with dispersion parameters $\nu_{i}<\text{1},$ ranging from 0.45 to 0.75. Scenario O1 combined small baseline and positive covariate-effect parameters with low dynamic persistence and spatial parameters τ=0.75and κ=0.30. Scenario O2 used moderate baseline and positive covariate-effect parameters, moderate dynamic persistence, and spatial parameters τ=1.50 and κ=0.50. Scenario O3 considered substantially larger baseline and positive covariate-effect parameters, moderate dynamic persistence, and spatial parameters τ=1.20 and κ=0.80. S2 Appendix (Tables S1–S3) presents the true parameter values, mean estimates, empirical biases, and mean absolute errors (MAEs) for Scenarios O1–O3 at time-series lengths N=500 and N=1000.

In Scenario O1 (S2 Appendix Table S1), the temporal parameters $\alpha_{i}$ and $\beta_{i}$ were estimated with relatively small biases, whereas the baseline, spatial, dispersion, and covariate-effect parameters generally exhibited positive biases. Increasing the time-series length fromN=500 to N=1000 generally reduced the MAEs, although the empirical biases did not decrease uniformly across all parameters. Scenario O2 (S2 Appendix Table S2) demonstrated strong overall estimation performance, with most mean estimates close to their true values, relatively small biases in the temporal, spatial, and dispersion parameters, and consistent reductions in MAE as the time-series length increased. In Scenario O3 (S2 Appendix Table S3), the temporal parameters $\alpha_{i}$and $\beta_{i},$spatial parameters τ and κ, and dispersion parameters νᵢ were estimated with relatively small errors. The larger absolute MAEs observed for the baseline parameters $\omega_{i}$ partly reflect the substantially larger true values considered in this scenario. However, the covariate-effect parameters $\phi_{i}$ remained positively biased at both time-series lengths. Moreover, increasing the time-series length did not consistently reduce the MAEs of the baseline and covariate-effect parameters.

Across the three overdispersion scenarios, the temporal parameters were generally estimated accurately under the different parameter configurations. Increasing the time-series length produced the clearest and most consistent improvements in Scenarios O1 and O2, particularly through reductions in MAE. The improvement was less uniform in Scenario O3, which involved substantially larger baseline and covariate-effect parameters. Overall, the conditional maximum likelihood estimator demonstrated satisfactory finite-sample performance for the identity-link SP-COM-INGARCHX (1 1) model under overdispersion, although estimation accuracy varied across parameters and scenarios.

#### 3.2.2 SP-COM-INGARCHX (1 1) underdispersion scenarios.

Three underdispersion scenarios were examined, with dispersion parameters $\nu_{i}>\text{1}$, ranging from 1.20 to 1.80. Scenario U1 combined small baseline and positive covariate-effect parameters with low dynamic persistence and spatial parametersτ=0.75 and κ=0.30. Scenario U2 used moderate baseline and positive covariate-effect parameters, moderate dynamic persistence, and spatial parameters τ=1.50 andκ=0.50. Scenario U3 is considered substantially larger baseline and positive covariate-effect parameters, moderate dynamic persistence, and spatial parameters τ=1.20and κ=0.80. S2 Appendix (Tables S4–S6) presents the true parameter values, mean estimates, empirical biases, and mean absolute errors (MAEs) for Scenarios U1–U3 at time-series lengthsN=500 and N=1000.

The underdispersion results revealed three distinct estimation patterns. In Scenario U1 (S2 Appendix Table S4), the baseline, temporal, and covariate-effect estimates closely tracked their true values, but τ and the dispersion parameters $\nu_{i}$ were systematically overestimated. Nevertheless, extending the series from N=500 to N=1000reduced the MAE of every parameter, indicating improved precision despite the remaining positive biases. Scenario U2 (S2 Appendix Table S5) provided the most balanced results: the temporal, spatial, and dispersion parameters showed little systematic bias, while every parameter exhibited a lower MAE at N=1000. The covariate-effect estimates were somewhat less precise than the dynamic and spatial estimates but still benefited from the longer series. Scenario U3 (S2 Appendix Table S6) presented a different pattern. The temporal, spatial, and dispersion components remained accurately estimated, but the substantially larger baseline and covariate-effect values were associated with larger absolute errors. In particular, the covariate effects were consistently overestimated, and the additional observations produced parameter-specific rather than uniform improvements.

Taken together, the results indicate that the temporal dynamics were estimated reliably across all three underdispersion settings. The gain from increasing the time-series length was especially evident in U1 and U2, whereas the large-scale parameter configuration in U3 created greater difficulty in estimating the baseline and covariate effects. Thus, the conditional maximum likelihood estimator showed satisfactory finite-sample performance for the identity-link SP-COM-INGARCHX (1,1) model under underdispersion, with its strongest performance observed for the temporal parameters and for the small-to-moderate parameter configurations.

### 3.3. Simulation study for the softplus-SP-COM-INGARCHX (1 1) model

Six scenarios were considered to evaluate the finite-sample performance of the conditional maximum likelihood estimator for the softplus SP-COM-INGARCHX (1,1) model. The softplus link ensures that the conditional COM-Poisson centering parameter remains positive while allowing negative covariate-effect coefficients. Unlike the identity-link SP-COM-INGARCHX simulations in Section 3.2, which used positive covariate-effect coefficients, the present simulations used $\phi_{i}<\text{0}$. The values of $\phi_{i}$ were selected to represent a wide range of coefficient magnitudes: small negative effects in O1 and U1 (−0.40 to −0.25), moderate negative effects in O2 and U2 (−3.50 to −2.00), and substantially larger negative effects in O3 and U3 (−340 to −200). This design allowed the estimator to be examined under different magnitudes of negative covariate effects. The overdispersion and underdispersion results are presented in Sections 3.3.1 and 3.3.2, respectively.

#### 3.3.1 Softplus SP-COM-INGARCHX (1 1) overdispersion scenarios.

This subsection presents the finite-sample estimation results for the three overdispersion scenarios, O1–O3, characterized by dispersion parameters ($\nu_{i}<\text{1}$). The negative covariate-effect coefficients ranged from −0.40 to −0.25 in O1, from −3.50 to −2.00 in O2, and from −340 to −200 in O3. Table 7, Table 8, Table 9 report the true parameter values, mean estimates, biases, and MAEs for time-series lengths N=500and N=1000.

**Table 7:** Softplus SP-COM-INGARCHX (1,1) simulation results under overdispersion: Scenario O1.

Parameter	Truth	Time Series Length N = 500			Time Series Length N = 1000		
		Mean Estimate	Bias	MAE	Mean Estimate	Bias	MAE
$\omega_{\text{1}}$	1.0000	1.2171	0.2171	0.2918	1.2047	0.2047	0.2455
$\omega_{\text{2}}$	1.3000	1.5242	0.2242	0.3282	1.5387	0.2387	0.2920
$\omega_{\text{3}}$	1.1000	1.4478	0.3478	0.3906	1.4692	0.3692	0.3855
$\omega_{\text{4}}$	1.5000	1.6511	0.1511	0.3258	1.6325	0.1325	0.2596
$\alpha_{\text{1}}$	0.1100	0.1038	−0.0062	0.0456	0.1038	−0.0062	0.0330
$\alpha_{\text{2}}$	0.0900	0.0872	−0.0028	0.0439	0.0839	−0.0061	0.0319
$\alpha_{\text{3}}$	0.1000	0.0954	−0.0046	0.0447	0.0945	−0.0055	0.0319
$\alpha_{\text{4}}$	0.0800	0.0755	−0.0045	0.0403	0.0770	−0.0030	0.0317
$\beta_{\text{1}}$	0.1900	0.2038	0.0138	0.1031	0.2073	0.0173	0.0796
$\beta_{\text{2}}$	0.2100	0.2258	0.0158	0.1118	0.2236	0.0136	0.0893
$\beta_{\text{3}}$	0.1800	0.2056	0.0256	0.0987	0.1955	0.0155	0.0726
$\beta_{\text{4}}$	0.2200	0.2256	0.0056	0.1182	0.2253	0.0053	0.0939
τ	0.7500	0.8233	0.0733	0.3547	0.8261	0.0761	0.2770
κ	0.3000	0.3523	0.0523	0.1270	0.3454	0.0454	0.1143
$\nu_{\text{1}}$	0.6500	0.9076	0.2576	0.2576	0.9036	0.2536	0.2536
$\nu_{\text{2}}$	0.5500	0.7556	0.2056	0.2056	0.7507	0.2007	0.2007
$\nu_{\text{3}}$	0.4500	0.6639	0.2139	0.2139	0.6608	0.2108	0.2108
$\nu_{\text{4}}$	0.7000	0.8868	0.1868	0.1869	0.8854	0.1854	0.1854
$\phi_{\text{1}}$	−0.3000	−0.2914	0.0086	0.1874	−0.2659	0.0341	0.1493
$\phi_{\text{2}}$	−0.3500	−0.3410	0.0090	0.1966	−0.3324	0.0176	0.1555
$\phi_{\text{3}}$	−0.2500	−0.2539	−0.0039	0.1715	−0.2283	0.0217	0.1315
$\phi_{\text{4}}$	−0.4000	−0.4052	−0.0052	0.2043	−0.3748	0.0252	0.1560

**Table 8:** Softplus SP-COM-INGARCHX (1,1) simulation results under overdispersion: Scenario O2.

Parameter	Truth	Time Series Length N = 500			Time Series Length N = 1000		
		Mean Estimate	Bias	MAE	Mean Estimate	Bias	MAE
$\omega_{\text{1}}$	10.0000	10.1185	0.1185	1.0657	10.0458	0.0458	0.7663
$\omega_{\text{2}}$	15.0000	15.0535	0.0535	1.5519	15.0670	0.0670	1.1508
$\omega_{\text{3}}$	12.0000	12.0068	0.0068	1.3133	11.9959	−0.0041	0.9461
$\omega_{\text{4}}$	17.0000	17.1480	0.1480	1.7332	17.1228	0.1228	1.3101
$\alpha_{\text{1}}$	0.2900	0.2849	−0.0051	0.0342	0.2879	−0.0021	0.0252
$\alpha_{\text{2}}$	0.3400	0.3341	−0.0059	0.0366	0.3368	−0.0032	0.0251
$\alpha_{\text{3}}$	0.2700	0.2655	−0.0045	0.0355	0.2656	−0.0044	0.0251
$\alpha_{\text{4}}$	0.3100	0.3040	−0.0060	0.0362	0.3066	−0.0034	0.0255
$\beta_{\text{1}}$	0.3100	0.3044	−0.0056	0.0527	0.3078	−0.0022	0.0353
$\beta_{\text{2}}$	0.2600	0.2594	−0.0006	0.0512	0.2589	−0.0011	0.0356
$\beta_{\text{3}}$	0.3300	0.3273	−0.0027	0.0504	0.3307	0.0007	0.0349
$\beta_{\text{4}}$	0.2900	0.2880	−0.0020	0.0524	0.2878	−0.0022	0.0375
τ	1.5000	1.5231	0.0231	0.0599	1.5215	0.0215	0.0513
κ	0.5000	0.5038	0.0038	0.0674	0.5029	0.0029	0.0487
$\nu_{\text{1}}$	0.6000	0.6121	0.0121	0.0327	0.6070	0.0070	0.0233
$\nu_{\text{2}}$	0.5000	0.5087	0.0087	0.0263	0.5066	0.0066	0.0184
$\nu_{\text{3}}$	0.4500	0.4581	0.0081	0.0240	0.4567	0.0067	0.0176
$\nu_{\text{4}}$	0.6000	0.6092	0.0092	0.0317	0.6072	0.0072	0.0231
$\phi_{\text{1}}$	−2.5000	−2.4648	0.0352	0.4774	−2.4828	0.0172	0.3351
$\phi_{\text{2}}$	−3.0000	−2.9310	0.0690	0.6041	−2.9706	0.0294	0.4342
$\phi_{\text{3}}$	−2.0000	−1.8325	0.1675	0.4289	−1.8671	0.1329	0.3382
$\phi_{\text{4}}$	−3.5000	−3.4305	0.0695	0.6539	−3.4480	0.0520	0.4767

**Table 9:** Softplus SP-COM-INGARCHX (1,1) simulation results under overdispersion: Scenario O3.

Parameter	Truth	Time Series Length N = 500			Time Series Length N = 1000		
		Mean Estimate	Bias	MAE	Mean Estimate	Bias	MAE
$\omega_{\text{1}}$	1000.0000	989.6857	−10.3143	22.5799	994.0690	−5.9310	20.4776
$\omega_{\text{2}}$	1500.0000	1490.6796	−9.3204	28.0872	1494.6398	−5.3602	27.5213
$\omega_{\text{3}}$	1200.0000	1191.1757	−8.8243	25.7530	1197.0534	−2.9466	24.2438
$\omega_{\text{4}}$	1700.0000	1687.6300	−12.3700	31.5359	1688.9321	−11.0679	31.3553
$\alpha_{\text{1}}$	0.2800	0.2818	0.0018	0.0048	0.2819	0.0019	0.0049
$\alpha_{\text{2}}$	0.3000	0.3012	0.0012	0.0052	0.3016	0.0016	0.0052
$\alpha_{\text{3}}$	0.3200	0.3220	0.0020	0.0052	0.3222	0.0022	0.0053
$\alpha_{\text{4}}$	0.2900	0.2921	0.0021	0.0054	0.2922	0.0022	0.0052
$\beta_{\text{1}}$	0.3200	0.3208	0.0008	0.0061	0.3204	0.0004	0.0065
$\beta_{\text{2}}$	0.3000	0.3017	0.0017	0.0061	0.3008	0.0008	0.0058
$\beta_{\text{3}}$	0.2800	0.2796	−0.0004	0.0069	0.2784	−0.0016	0.0068
$\beta_{\text{4}}$	0.3100	0.3105	0.0005	0.0060	0.3104	0.0004	0.0058
τ	1.2000	1.2068	0.0068	0.0325	1.2135	0.0135	0.0381
κ	0.8000	0.7917	−0.0083	0.0150	0.7922	−0.0078	0.0149
$\nu_{\text{1}}$	0.7500	0.7457	−0.0043	0.0170	0.7444	−0.0056	0.0169
$\nu_{\text{2}}$	0.7000	0.6957	−0.0043	0.0178	0.6954	−0.0046	0.0182
$\nu_{\text{3}}$	0.6000	0.5970	−0.0030	0.0190	0.5962	−0.0038	0.0199
$\nu_{\text{4}}$	0.7000	0.6955	−0.0045	0.0201	0.6950	−0.0050	0.0209
$\phi_{\text{1}}$	−200.0000	−173.7058	26.2942	34.6518	−177.5160	22.4840	31.7103
$\phi_{\text{2}}$	−300.0000	−279.4247	20.5753	33.4280	−283.2947	16.7053	29.5853
$\phi_{\text{3}}$	−240.0000	−207.8415	32.1585	37.6904	−207.8783	32.1217	37.7847
$\phi_{\text{4}}$	−340.0000	−318.3337	21.6663	30.0028	−318.6722	21.3278	30.6048

The spatial and temporal dependence parameters were generally estimated accurately across O1-O3. Increasing the time-series length generally improved their estimation in O1 and O2, whereas their errors were already very small in O3 and remained stable as N increased. The spatial parameters τand κ were less precisely estimated in O1, particularly τ, but were recovered more accurately in O2 and O3. A similar pattern was observed for the dispersion parameters $\nu_{i}$: they were systematically overestimated toward one in O1, with little reduction in bias at N=1000, but showed relatively small biases and MAEs in O2 and O3. The baseline parameters $\omega_{i}$ exhibited positive biases in O1 were estimated more accurately in O2, and showed larger absolute MAEs in O3. However, the O3 errors were small relative to the true baseline values of 1,000–1,700. Thus, as the baseline parameter scale increased from O1 to O3, the dynamic, spatial, and dispersion components were generally estimated more accurately, although the scenarios also differed in other parameter settings.

All negative covariate-effect coefficients were recovered with the correct sign across the three scenarios. The small negative $\phi_{i}$ values in O1 were estimated close to their true values, with MAEs generally decreasing as N increased. The moderate negative effects in O2 were also reasonably estimated, although some estimates, particularly $\phi_{\text{3}}$, were attenuated toward zero. This attenuation was more evident in O3, where the substantially larger negative effects produced larger absolute biases and MAEs, despite remaining correctly signed. These errors should be interpreted relative to the much larger magnitudes of the true O3 coefficients. Overall, increasing N generally reduced bias and MAE, particularly in O1 and O2, but the improvement was not uniform across all parameters in O3. The conditional maximum likelihood estimation method therefore demonstrated satisfactory finite-sample accuracy for the softplus SP-COM-INGARCHX (1 1) model, while estimation remained more challenging for the spatial and dispersion parameters in O1 and the very large negative covariate effects in O3.

#### 3.3.2 Softplus SP-COM-INGARCHX (1 1) Underdispersion Scenarios.

This subsection presents the finite-sample estimation results for the three underdispersion scenarios, U1–U3, characterized by dispersion parameters $\nu_{i}>\text{1}$. The negative covariate-effect coefficients ranged from −0.40 to −0.25 in U1, from −3.50 to −2.00 in U2, and from −340 to −200 in U3. Table 10, Table 11, Table 12 report the true parameter values, mean estimates, biases, and MAEs for time-series lengths N=500and N=1000.

**Table 10:** Softplus SP-COM-INGARCHX (1 1) simulation results under underdispersion: Scenario U1.

Parameter	Truth	Time Series Length N = 500			Time Series Length N = 1000		
		Mean Estimate	Bias	MAE	Mean Estimate	Bias	MAE
$\omega_{\text{1}}$	1.0000	1.0499	0.0499	0.1518	1.0366	0.0366	0.1103
$\omega_{\text{2}}$	1.4000	1.4232	0.0232	0.1987	1.4191	0.0191	0.1310
$\omega_{\text{3}}$	1.2000	1.2116	0.0116	0.1750	1.2058	0.0058	0.1112
$\omega_{\text{4}}$	1.6000	1.6349	0.0349	0.1929	1.6227	0.0227	0.1295
$\alpha_{\text{1}}$	0.1200	0.1158	−0.0042	0.0484	0.1191	−0.0009	0.0352
$\alpha_{\text{2}}$	0.1000	0.0962	−0.0038	0.0438	0.0963	−0.0037	0.0312
$\alpha_{\text{3}}$	0.0800	0.0768	−0.0032	0.0394	0.0804	0.0004	0.0301
$\alpha_{\text{4}}$	0.1100	0.1055	−0.0045	0.0452	0.1076	−0.0024	0.0309
$\beta_{\text{1}}$	0.1800	0.1892	0.0092	0.0639	0.1916	0.0116	0.0449
$\beta_{\text{2}}$	0.2000	0.1995	−0.0005	0.0826	0.2037	0.0037	0.0530
$\beta_{\text{3}}$	0.2200	0.2220	0.0020	0.0852	0.2238	0.0038	0.0539
$\beta_{\text{4}}$	0.1700	0.1710	0.0010	0.0685	0.1726	0.0026	0.0463
τ	0.7500	0.9652	0.2152	0.3698	0.9663	0.2163	0.3335
κ	0.3000	0.3388	0.0388	0.0985	0.3340	0.0340	0.0807
$\nu_{\text{1}}$	1.3000	1.6100	0.3100	0.3100	1.6014	0.3014	0.3014
$\nu_{\text{2}}$	1.5000	1.7134	0.2134	0.2151	1.7106	0.2106	0.2107
$\nu_{\text{3}}$	1.7000	1.9572	0.2572	0.2586	1.9486	0.2486	0.2486
$\nu_{\text{4}}$	1.4000	1.5913	0.1913	0.1933	1.5799	0.1799	0.1802
$\phi_{\text{1}}$	−0.3000	−0.3085	−0.0085	0.1487	−0.2963	0.0037	0.1053
$\phi_{\text{2}}$	−0.3500	−0.3496	0.0004	0.1447	−0.3514	−0.0014	0.1037
$\phi_{\text{3}}$	−0.2500	−0.2562	−0.0062	0.1247	−0.2565	−0.0065	0.0866
$\phi_{\text{4}}$	−0.4000	−0.4112	−0.0112	0.1604	−0.4042	−0.0042	0.1130

**Table 11:** Softplus SP-COM-INGARCHX (1,1) Simulation Results Under Underdispersion: Scenario U2.

Parameter	Truth	Time Series Length N = 500			Time Series Length N = 1000		
		Mean Estimate	Bias	MAE	Mean Estimate	Bias	MAE
$\omega_{\text{1}}$	10.0000	9.9889	−0.0111	0.7559	10.0039	0.0039	0.5412
$\omega_{\text{2}}$	15.0000	15.0356	0.0356	1.0605	15.0460	0.0460	0.7967
$\omega_{\text{3}}$	12.0000	11.9868	−0.0132	0.8774	11.9701	−0.0299	0.6138
$\omega_{\text{4}}$	17.0000	17.0640	0.0640	1.1886	17.0817	0.0817	0.8854
$\alpha_{\text{1}}$	0.3200	0.3149	−0.0051	0.0281	0.3176	−0.0024	0.0201
$\alpha_{\text{2}}$	0.2800	0.2763	−0.0037	0.0284	0.2787	−0.0013	0.0209
$\alpha_{\text{3}}$	0.3500	0.3458	−0.0042	0.0278	0.3492	−0.0008	0.0201
$\alpha_{\text{4}}$	0.3000	0.2953	−0.0047	0.0276	0.2976	−0.0024	0.0200
$\beta_{\text{1}}$	0.2800	0.2803	0.0003	0.0283	0.2802	0.0002	0.0197
$\beta_{\text{2}}$	0.3300	0.3299	−0.0001	0.0286	0.3287	−0.0013	0.0211
$\beta_{\text{3}}$	0.2500	0.2487	−0.0013	0.0251	0.2488	−0.0012	0.0167
$\beta_{\text{4}}$	0.3100	0.3104	0.0004	0.0289	0.3087	−0.0013	0.0202
τ	1.5000	1.5201	0.0201	0.0531	1.5154	0.0154	0.0478
κ	0.5000	0.5085	0.0085	0.0470	0.5034	0.0034	0.0325
$\nu_{\text{1}}$	1.4000	1.4216	0.0216	0.0575	1.4153	0.0153	0.0392
$\nu_{\text{2}}$	1.6000	1.6192	0.0192	0.0699	1.6099	0.0099	0.0488
$\nu_{\text{3}}$	1.8000	1.8277	0.0277	0.0878	1.8176	0.0176	0.0631
$\nu_{\text{4}}$	1.5000	1.5191	0.0191	0.0664	1.5082	0.0082	0.0448
$\phi_{\text{1}}$	−2.5000	−2.4785	0.0215	0.2794	−2.4784	0.0216	0.2145
$\phi_{\text{2}}$	−3.0000	−2.9882	0.0118	0.3490	−2.9789	0.0211	0.2578
$\phi_{\text{3}}$	−2.0000	−1.8858	0.1142	0.2922	−1.9024	0.0976	0.2263
$\phi_{\text{4}}$	−3.5000	−3.4947	0.0053	0.4079	−3.4548	0.0452	0.2930

**Table 12:** Softplus SP-COM-INGARCHX (1,1) Simulation Results Under Underdispersion: Scenario U3.

Parameter	Truth	Time Series Length N = 500			Time Series Length N = 1000		
		Mean Estimate	Bias	MAE	Mean Estimate	Bias	MAE
$\omega_{\text{1}}$	1000.0000	990.9174	−9.0826	20.8342	992.8332	−7.1668	19.8964
$\omega_{\text{2}}$	1500.0000	1488.0571	−11.9429	28.1463	1491.7695	−8.2305	29.0412
$\omega_{\text{3}}$	1200.0000	1187.8325	−12.1675	24.2749	1190.7550	−9.2450	25.5149
$\omega_{\text{4}}$	1700.0000	1688.3490	−11.6510	29.1481	1688.9728	−11.0272	32.1413
$\alpha_{\text{1}}$	0.2700	0.2718	0.0018	0.0045	0.2719	0.0019	0.0049
$\alpha_{\text{2}}$	0.3100	0.3113	0.0013	0.0048	0.3113	0.0013	0.0049
$\alpha_{\text{3}}$	0.2900	0.2926	0.0026	0.0051	0.2927	0.0027	0.0052
$\alpha_{\text{4}}$	0.3300	0.3325	0.0025	0.0053	0.3322	0.0022	0.0055
$\beta_{\text{1}}$	0.3300	0.3306	0.0006	0.0062	0.3306	0.0006	0.0063
$\beta_{\text{2}}$	0.2900	0.2918	0.0018	0.0058	0.2916	0.0016	0.0059
$\beta_{\text{3}}$	0.3200	0.3199	−0.0001	0.0065	0.3193	−0.0007	0.0067
$\beta_{\text{4}}$	0.2700	0.2701	0.0001	0.0058	0.2706	0.0006	0.0061
τ	1.2000	1.2107	0.0107	0.0331	1.2114	0.0114	0.0338
κ	0.8000	0.7921	−0.0079	0.0154	0.7908	−0.0092	0.0158
$\nu_{\text{1}}$	1.2000	1.2005	0.0005	0.0222	1.1988	−0.0012	0.0216
$\nu_{\text{2}}$	1.2500	1.2483	−0.0017	0.0256	1.2482	−0.0018	0.0242
$\nu_{\text{3}}$	1.3000	1.2990	−0.0010	0.0280	1.2968	−0.0032	0.0274
$\nu_{\text{4}}$	1.2000	1.2004	0.0004	0.0222	1.1993	−0.0007	0.0224
$\phi_{\text{1}}$	−200.0000	−173.6857	26.3143	34.8491	−173.9218	26.0782	34.6698
$\phi_{\text{2}}$	−300.0000	−275.8839	24.1161	35.3762	−275.7655	24.2345	36.7160
$\phi_{\text{3}}$	−240.0000	−211.7548	28.2452	33.4353	−209.6036	30.3964	35.4653
$\phi_{\text{4}}$	−340.0000	−315.9798	24.0202	32.5872	−313.6348	26.3652	36.2870

The spatial and temporal dependence parameters were generally estimated accurately across U1–U3. The $\alpha_{i}$ parameters, representing the effects of spatially weighted lagged observations, and the $\beta_{i}$ parameters, measuring temporal persistence, showed relatively small biases and MAEs. Increasing N generally reduced their MAEs in U1 and U2, whereas their errors were already very small in U3 and remained relatively stable. The spatial decay parameters τand κwere less precisely estimated in U1, particularlyτ, but were recovered more accurately in U2 and U3. The dispersion parameters $\nu_{i}$were systematically overestimated in U1, and their biases persisted when N increased. In contrast, the dispersion parameters in U2 and U3 were estimated with relatively small biases and MAEs. The baseline parameters ωᵢ showed moderate positive biases in U1 and were estimated more accurately in U2. Although their absolute errors were larger in U3, these errors were small relative to the much larger true baseline values of 1,000–1,700. Thus, across the scenario configurations, the temporal, spatial, and dispersion parameters were generally estimated more accurately in U2 and U3 than in U1, while the absolute errors of the baseline estimates increased with their parameter scale.

The mean estimates of all negative covariate-effect coefficients retained the correct sign. The small negative$\phi_{i}$values in U1 were estimated close to their true values, and their MAEs decreased as N increased. The moderate negative effects in U2 were also estimated reasonably accurately. In U3, the substantially larger negative effects remained correctly signed but were consistently estimated as less negative than their true values, resulting in larger absolute biases and MAEs. These errors should be interpreted relative to the considerably larger magnitudes of the true U3 coefficients. Overall, increasing N generally reduced MAE in U1 and U2, although reductions in bias were not uniform. In U3, the estimation errors remained stable or increased slightly for several parameters. The conditional maximum likelihood estimator therefore demonstrated generally satisfactory finite-sample accuracy under underdispersion, with the principal estimation challenges involving the spatial and dispersion parameters in U1 and the magnitudes of the very large negative covariate effects in U3.

## 4. Real data example

To evaluate their empirical performance, the proposed SP-COM-INGARCH, SP-COM-INGARCHX, and softplus SP-COM-INGARCHX models were applied to weekly influenza-like illness (ILI) counts from Arkansas (AR), Kansas (KS), Missouri (MO), and Oklahoma (OK). The corresponding 2020 mean centers of population, reported as latitude and longitude, were AR (35.199251, −92.713212), KS (38.480984, −96.409286), MO (38.432921, −92.234929), and OK (35.606866, −96.854171). Weekly absolute humidity, obtained as described in Section 2.2, was included as an exogenous covariate in the INGARCHX models. Absolute humidity was selected because previous studies have shown that low absolute humidity enhances influenza virus survival and transmission and is associated with the seasonal onset of influenza epidemics [25, 26]. For comparison, the corresponding spatial generalized Poisson models were fitted to the same data: SP-COM-INGARCH was compared with SP-GP-INGARCH, SP-COM-INGARCHX with SP-GP-INGARCHX, and softplus SP-COM-INGARCHX with softplus SP-GP-INGARCHX.

The initial in-sample period comprised 699 weeks, from MMWR week 40 of 2010 through MMWR week 8 of 2024. In this study, the COVID-19 period was operationally defined as MMWR week 11 of 2020 through MMWR week 19 of 2023. These boundaries correspond to the weeks containing the WHO’s characterization of COVID-19 as a pandemic on March 11, 2020, and the expiration of the U.S. COVID-19 public health emergency on May 11, 2023, respectively [42, 43]. In-sample model fit was evaluated using the log-likelihood (LLF), Akaike information criterion (AIC), and Bayesian information criterion (BIC), with higher LLF and lower AIC and BIC values indicating better fit. In-sample prediction accuracy was evaluated using the root mean squared error (RMSE) and mean absolute error (MAE), calculated for both the overall in-sample period and the COVID-19 period. Prediction-interval calibration was assessed using the percentage of observed counts falling outside the 95% prediction intervals.

The subsequent 52 weeks, from MMWR week 9 of 2024 through MMWR week 8 of 2025, were reserved for rolling one-step-ahead out-of-sample forecasting. This forecasting window allowed predictive performance to be evaluated over a full annual seasonal cycle, including both influenza-season and off-season activity. Out-of-sample forecast performance was evaluated using RMSE, MAE, and the percentage of observed counts falling outside the 95% prediction intervals.

Tables 13 and 14 summarize weekly ILI counts and absolute humidity, respectively, across the full, in-sample, and out-of-sample periods. All four states exhibited pronounced overdispersion, while zero-count weeks remained below 5% over the full study period. Highest dispersion is observed in OK for both the in- and out-of-sample periods. Mean ILI counts were higher during the out-of-sample period, consistent with the elevated baseline levels and several large peaks observed in the later years in Fig 1. It also shows a noticeable upward shift during the latter part of the COVID-19 period: seasonal troughs and other lower-range counts were elevated relative to the pre-COVID-19 period, and several pronounced peaks occurred. Absolute humidity displayed a strong annual cycle, with periods of lower humidity generally coinciding with higher ILI activity across the four states. This visual pattern is consistent with the inverse relationship reported in the literature, whereby low absolute humidity enhances influenza virus survival and airborne transmission, supporting its inclusion as an exogenous covariate.

**Table 13:** Descriptive statistics of weekly influenza-like illness counts by state and study period.

Period	State	Mean	Variance	Minimum	Median	Maximum	Dispersion Ratio	Zero weeks (%)
Full Period	KS	183.82	54112.86	0	99	1448	294.37	0.67
	OK	200.74	75821.56	0	109	2219	377.72	1.07
	MO	210.4	68143.66	0	132	2038	323.88	1.6
	AR	158.18	38153.52	0	97	1392	241.2	3.2
In sample	KS	172.31	49601.21	0	92	1448	287.87	0.72
	OK	181.93	63611.16	0	93	1953	349.65	1.14
	MO	196.41	62617.51	0	113	2038	318.81	1.72
	AR	145.56	35930.67	0	81	1392	246.85	3.43
Out of Sample	KS	338.65	90661.09	50	222.5	1198	267.71	0
	OK	453.6	174381.62	100	287	2219	384.44	0
	MO	398.38	106398.79	118	267.5	1516	267.08	0
	AR	327.87	37783.37	107	264.5	951	115.24	0

**Table 14:** Descriptive statistics of weekly absolute humidity by state and study period.

Period	State	Mean	Variance	Minimum	Median	Maximum
Full Period	KS	8.59	23.6	1.51	7.36	20.06
	OK	10.15	25.73	1.75	9.02	21.41
	MO	9.88	27.64	1.47	8.52	22.45
	AR	11.65	27.07	2.31	10.77	22.07
In sample	KS	8.59	23.83	1.51	7.33	20.06
	OK	10.12	25.92	1.75	8.94	21.41
	MO	9.86	27.82	1.47	8.51	22.45
	AR	11.63	27.27	2.31	10.74	22.07
Out of Sample	KS	8.6	21.01	1.63	8.02	17.36
	OK	10.5	23.5	2.19	10.18	18.9
	MO	10.1	25.68	1.92	9.28	19.24
	AR	11.89	24.83	3	11.5	19.6

**Note**: Absolute humidity is reported in grams per cubic meter (g/m^3^)

**Fig 1:**
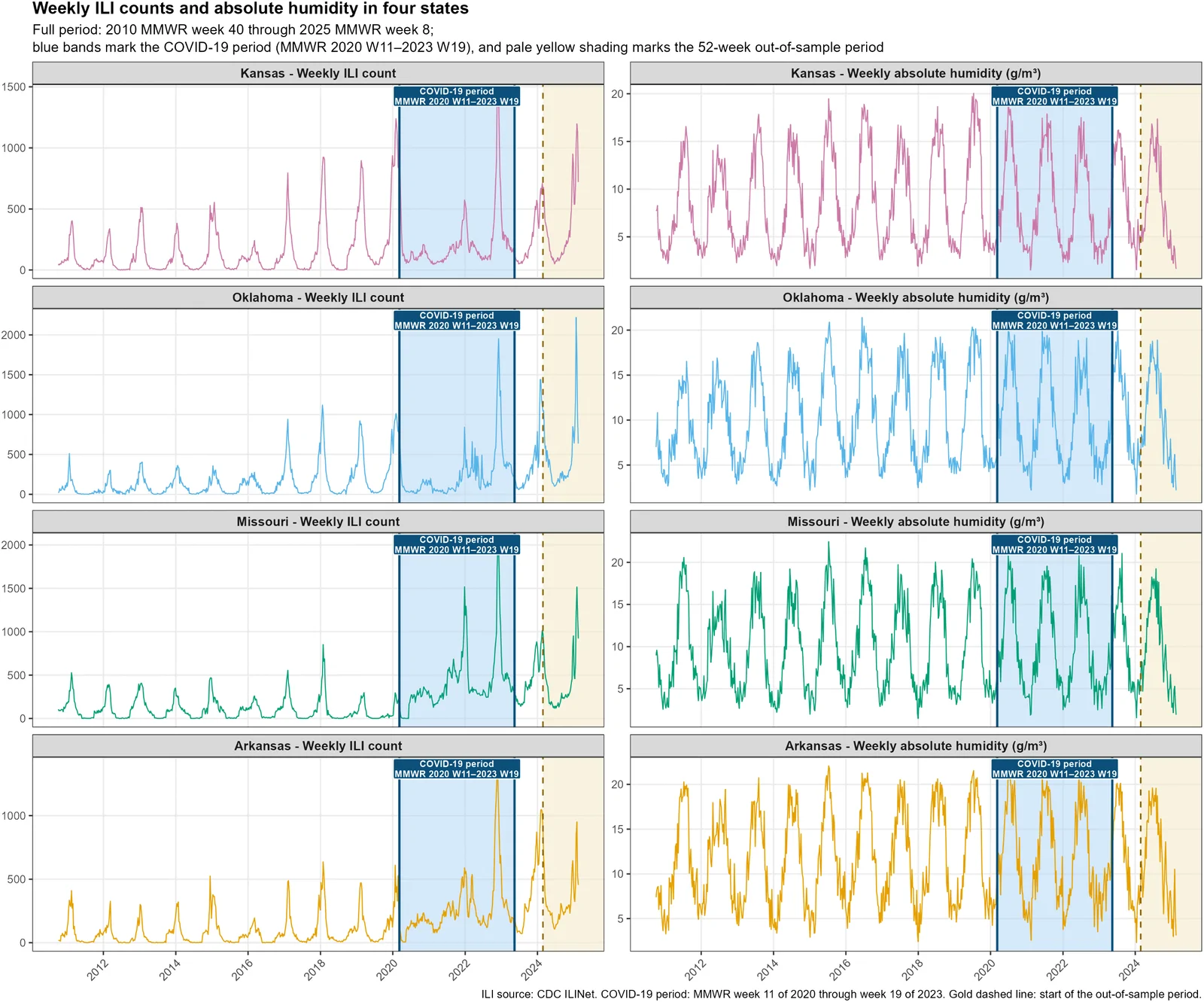
Weekly Influenza-Like Illness Counts and Absolute Humidity in Arkansas, Kansas, Missouri, and Oklahoma.

### 4.1. In-sample estimation

Table 15 summarizes the in-sample performance of the SP-COM-INGARCH (1,1) (**M1**), identity-link SP-COM-INGARCHX (1,1) (**M2**), and softplus SP-COM-INGARCHX (1,1) (**M3**), both by state and jointly where applicable. Overall, M3 provided the strongest likelihood-based fit, with the highest joint log-likelihood and the lowest joint AIC and BIC. At the state level, M3 also had the lowest AIC for all four states and the highest log-likelihood for each state; its BIC was lowest for Arkansas, Missouri, and Oklahoma, whereas M1 had the lowest BIC for Kansas. Notably, M3 performed particularly well for Oklahoma, the state exhibiting the greatest dispersion, with lower in-sample RMSE and MAE than M1 and M2. The percentage of observations falling outside the 95% prediction intervals ranged from 5.02% to 6.89% across the three models and four states, remaining reasonably close to the nominal 5% rate, although values above 5% indicate slight under coverage. M3 achieved the value closest to the nominal level in Missouri (5.02%) and also improved interval performance in Oklahoma relative to M1 and M2.

**Table 15:** In-sample model fit and prediction accuracy of SP-COM-INGARCH models for weekly influenza-like illness counts, including accuracy during the COVID-19 period, by state.

Model	Evaluation Measure	AR	KS	MO	OK	Total
M1	AIC	6585.64	6679.48	6802.61	7204.83	27164.55
	BIC	6692.46	**6786.30**	6909.43	7311.65	27271.37
	LLF	−3274.82	−3321.74	−3383.30	−3584.41	−13564.28
	Outside 95% Prediction Interval (%)	**6.60%**	**5.59%**	**6.02%**	6.89%	–
	RMSE (In-sample)	57.03	59.31	**69.48**	89.24	139.86
	MAE (In-sample)	32.55	33.11	36.69	50.01	152.35
	RMSE (COVID-19)	70.99	78.52	**113.97**	122.76	198.16
	MAE (COVID-19)	44.79	42.15	**62.92**	71.83	221.69
M2	AIC	6591.56	6687.12	6811.09	7207.74	27165.52
	BIC	6722.12	6817.68	6941.65	7338.30	27296.08
	LLF	−3273.78	−3321.56	−3383.55	−3581.87	−13560.76
	Outside 95% Prediction Interval (%)	6.60%	5.59%	6.02%	6.89%	–
	RMSE (In-sample)	57.03	**59.31**	69.48	89.36	139.94
	MAE (In-sample)	32.53	**33.10**	36.68	50.08	152.39
	RMSE (COVID-19)	**70.99**	**78.52**	113.97	122.76	**198.15**
	MAE (COVID-19)	**44.79**	**42.15**	62.92	71.75	**221.60**
M3	AIC	**6549.65**	**6678.78**	**6763.92**	**7149.51**	**27003.86**
	BIC	**6686.15**	6815.28	**6900.41**	**7286.00**	**27140.36**
	LLF	**−3251.83**	**−3316.39**	**−3358.96**	**−3551.76**	**−13478.93**
	Outside 95% Prediction Interval (%)	6.89%	6.03%	**5.02%**	**6.74%**	–
	RMSE (In-sample)	57.16	59.59	69.48	**87.48**	**138.92**
	MAE (In-sample)	**32.37**	33.94	**36.31**	**48.41**	**151.03**
	RMSE (COVID-19)	73.09	78.99	114.02	**122.06**	198.69
	MAE (COVID-19)	46.40	44.83	64.02	**71.70**	226.94

**Note:** Bold values indicate the best-performing value within each state/Total column for the corresponding evaluation measure across M1-M3.

In terms of point-prediction accuracy, M3 achieved the lowest overall joint in-sample RMSE (138.92) and MAE (151.03), supporting its stronger performance over the full estimation period. During the COVID-19 period, however, M2 produced the lowest joint RMSE (198.15) and MAE (221.60), although these improvements over M1 were marginal and the three models produced broadly similar COVID-19 period RMSE values. The comparatively limited improvement from M1 to the identity-link M2 is consistent with the restriction imposed by the identity formulation when negative covariate effects must be accommodated while maintaining a positive conditional centering parameter. In contrast, the softplus formulation in M3 allows the absolute-humidity effect to be either positive or negative while preserving positivity of the conditional centering parameter. Taken together, the likelihood criteria, overall, in-sample prediction accuracy, and prediction-interval performance support M3 as the preferred SP-COM-INGARCH specification.

The state-specific in-sample fit of the three SP-COM-INGARCH models is further illustrated in Fig 2, which compares the observed weekly ILI counts with the corresponding fitted conditional means and 95% prediction intervals for Arkansas, Kansas, Missouri, and Oklahoma. The figure also highlights the COVID-19 period, allowing visual assessment of model performance during the major disruption in influenza-like illness patterns.

**Fig 2:**
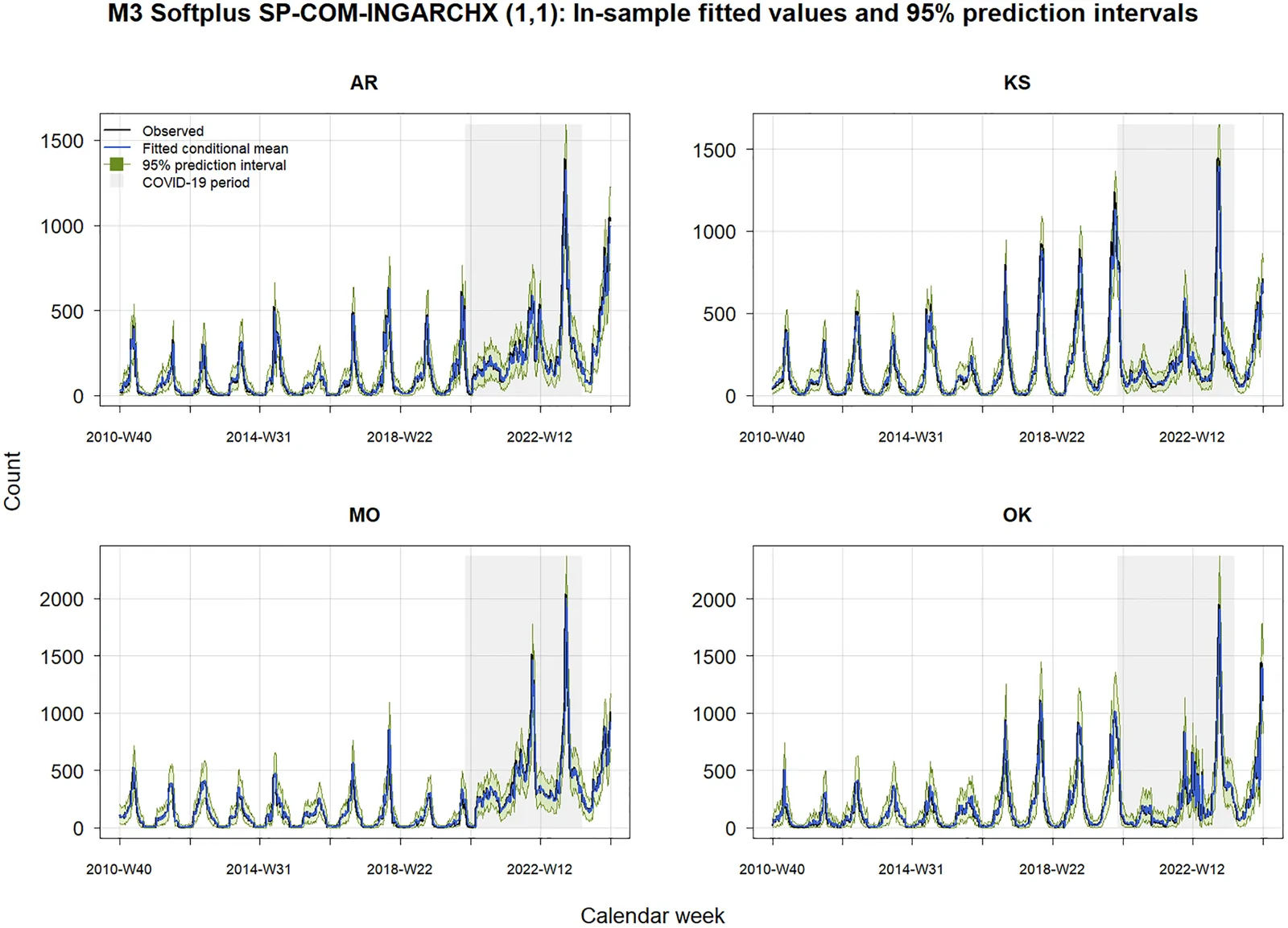
In-sample fitted weekly influenza-like illness counts and 95% prediction intervals from the softplus SP-COM-INGARCHX (1 1) model (M3) for Arkansas, Kansas, Missouri, and Oklahoma.

**Table 16** reports the in-sample results for the SP-GP-INGARCH (1 1) (**M4**), SP-GP-INGARCHX (1 1) (**M5**), and softplus SP-GP-INGARCHX (1 1) (**M6**). Based on the information criteria, M4 was the most favorable specification, with the lowest AIC and BIC both jointly and for each state. M6, however, attained the highest joint log-likelihood and the highest state-specific log-likelihoods for Arkansas, Kansas, and Oklahoma, whereas M5 had the highest log-likelihood for Missouri. These results suggest that the additional humidity and softplus terms improved the likelihood only modestly, and the gain was not large enough to offset the additional model complexity under AIC and BIC.

**Table 16:** In-sample model fit and prediction accuracy of SP-GP-INGARCH models for weekly influenza-like illness counts, including accuracy during the COVID-19 period, by state.

Model	Evaluation Measure	AR	KS	MO	OK	Total
M4	AIC	**6467.60**	**6602.99**	**6689.66**	**7143.39**	**26795.64**
	BIC	**6574.42**	**6709.81**	**6796.48**	**7250.21**	**26902.46**
	LLF	−3215.80	−3283.49	−3326.83	−3553.70	−13379.82
	Outside 95% Prediction Interval (%)	**5.74%**	**5.16%**	5.88%	**5.16%**	–
	RMSE (In-sample)	57.49	59.44	69.34	**87.38**	**138.86**
	MAE (In-sample)	32.17	32.91	36.20	48.83	150.10
	RMSE (COVID-19)	73.27	79.73	113.64	**120.84**	**198.09**
	MAE (COVID-19)	45.95	43.02	63.00	**71.03**	223.00
M5	AIC	6474.78	6611.59	6694.77	7153.17	26802.32
	BIC	6605.34	6742.15	6825.32	7283.73	26932.87
	LLF	−3215.39	−3283.80	**−3325.38**	−3554.59	−13379.16
	Outside 95% Prediction Interval (%)	6.17%	5.45%	**5.45%**	5.31%	–
	RMSE (In-sample)	**57.27**	**59.37**	69.41	87.56	138.88
	MAE (In-sample)	**32.03**	**32.84**	**36.17**	**48.81**	**149.85**
	RMSE (COVID-19)	**72.33**	**79.42**	113.87	121.55	198.19
	MAE (COVID-19)	**45.43**	**42.74**	63.08	71.38	**222.63**
M6	AIC	6475.83	6612.22	6701.48	7151.28	26802.80
	BIC	6612.32	6748.71	6837.97	7287.77	26939.29
	LLF	**−3214.91**	**−3283.11**	−3327.74	**−3552.64**	**−13378.40**
	Outside 95% Prediction Interval (%)	6.03%	5.45%	5.74%	5.31%	–
	RMSE (In-sample)	57.37	59.45	**69.30**	87.56	138.91
	MAE (In-sample)	32.09	32.89	36.22	48.96	150.16
	RMSE (COVID-19)	73.00	79.79	**113.55**	121.14	198.15
	MAE (COVID-19)	45.80	43.13	**62.99**	71.47	223.38

**Note:** Bold values indicate the best-performing value within each state/Total column for the corresponding evaluation measure across M4-M6.

Prediction-interval performance was generally close to the nominal level. The proportion of observations outside the 95% prediction intervals ranged from 5.16% to 6.17%. M4 was closest to the nominal 5% rate in Arkansas, Kansas, and Oklahoma, while M5 was closest in Missouri. Point-prediction accuracy was also very similar across the three models. For overall, in-sample RMSE, M5 had the lowest values in Arkansas and Kansas, M6 in Missouri, and M4 in Oklahoma; M4 also had the lowest joint RMSE (138.86). For MAE, M5 had the lowest values across all four states and the lowest joint MAE (149.85). During the COVID-19 period, the best-performing model varied by state: M5 had the lowest RMSE in Arkansas and Kansas, M6 in Missouri, and M4 in Oklahoma. At the joint level, M4 had the lowest COVID-period RMSE (198.09), whereas M5 had the lowest COVID-period MAE (222.63).

Overall, the three GP models showed very similar in-sample predictive accuracy. M4 was favored by AIC, BIC, interval calibration, and joint RMSE, while M5 showed a modest advantage in MAE. These results indicate that the added covariate and softplus terms offered only limited in-sample improvement within the GP family.

The state-specific in-sample fit of the selected GP specification is shown in Fig 3, where the observed weekly ILI counts are compared with the fitted conditional means and corresponding 95% prediction intervals for Arkansas, Kansas, Missouri, and Oklahoma. The COVID-19 period is also highlighted to illustrate how the SP-GP-INGARCH model tracked the marked changes in ILI activity during that interval.

**Fig 3:**
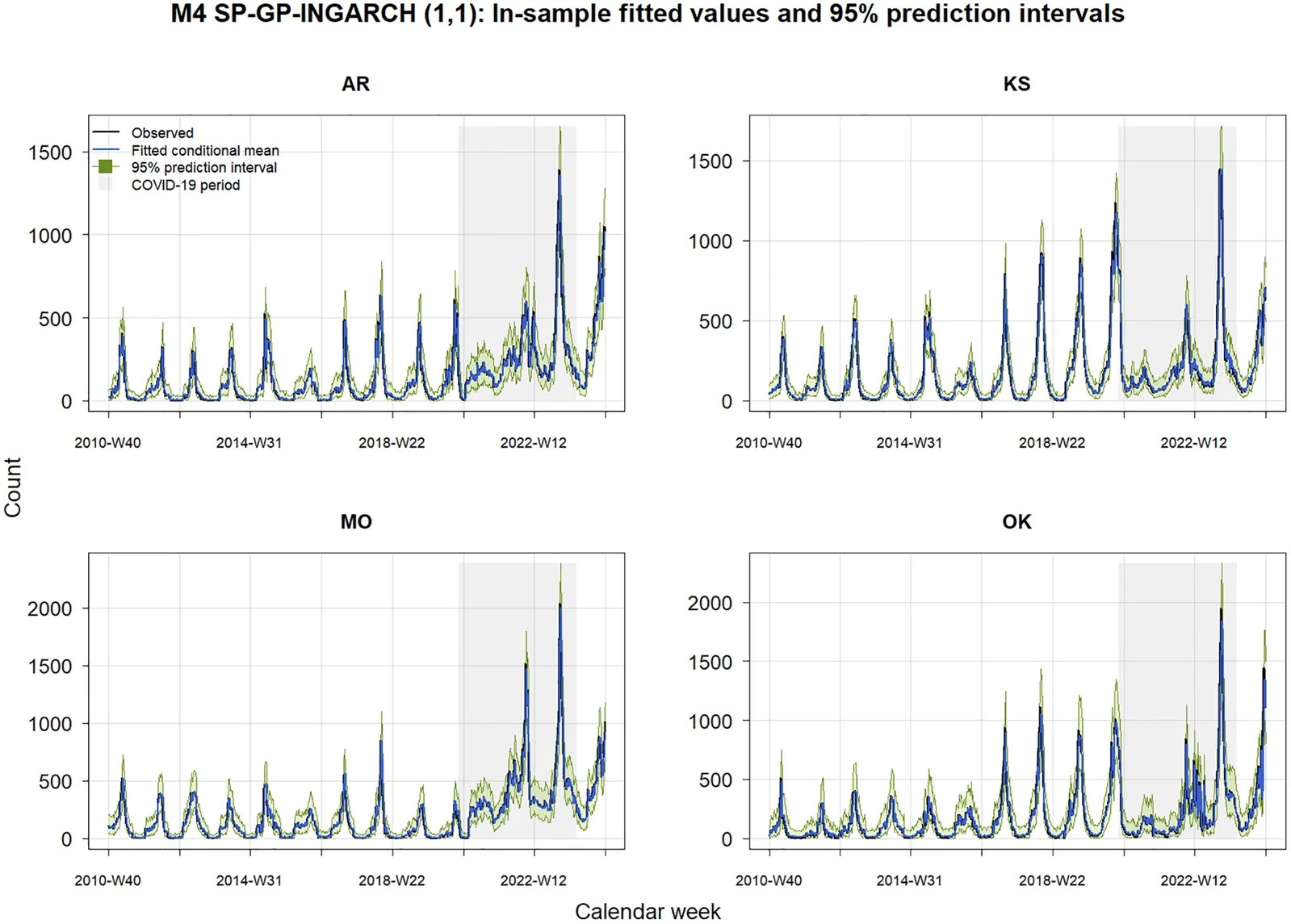
In-sample fitted weekly influenza-like illness counts and 95% prediction intervals from the SP-GP-INGARCH (1 1) model (M4) for Arkansas, Kansas, Missouri, and Oklahoma.

Residual analyses were conducted, and Pearson residuals were generally centered near zero across all six models, with M3 showing the closest overall centering among the COM-Poisson models. Of the 2,796 in-sample state-week residuals evaluated for each model, 165 (5.90%), 168 (6.01%), and 174 (6.22%) for M1, M2, and M3, respectively, fell outside the approximate 95% standard-normal reference band (−1.96, 1.96). For comparison, 142 (5.08%), 142 (5.08%), and 140 (5.01%) for M4, M5, and M6, respectively, fell outside the same reference band. These findings indicate broadly comparable residual exceedance rates, with the generalized Poisson models showing rates closer to the 5% standard-normal reference rate.

### 4.2. Out-of-Sample Estimation

Table 17 presents the out-of-sample forecasting performance of all six models over the 52-week forecasting period using RMSE, MAE, and the percentage of observations falling outside the 95% prediction intervals. The softplus SP-COM-INGARCHX model (M3) achieved the lowest joint RMSE (302.96), followed very closely by M1 (302.98). Among the generalized Poisson models, the joint RMSEs ranged from 303.52 to 303.56, indicating broadly comparable forecasting performance, although none improved upon the joint RMSE obtained by M3. In contrast, M1 produced the lowest joint MAE (321.78). At the state level, M3 had the lowest RMSE for Arkansas and Kansas, M1 for Missouri, and M5 for Oklahoma, showing that no single model dominated across all states and error measures.

**Table 17:** Out-of-sample forecasting performance over the 52-week forecast period for all six SP-INGARCH models, by state.

Model	Evaluation Measure	AR	KS	MO	OK	Total
M1	Outside 95% Prediction Interval (%)	7.69%	13.46%	9.62%	9.62%	–
	RMSE	105.46	109.65	**144.93**	218.28	302.98
	MAE	**63.14**	65.58	**80.95**	**112.10**	**321.78**
M2	Outside 95% Prediction Interval (%)	7.69%	13.46%	9.62%	9.62%	–
	RMSE	105.74	109.51	145.56	220.60	305.00
	MAE	63.44	65.56	81.66	112.59	323.24
M3	Outside 95% Prediction Interval (%)	9.62%	13.46%	7.69%	9.62%	–
	RMSE	**104.65**	**108.19**	145.45	219.03	**302.96**
	MAE	63.55	**64.82**	82.52	115.63	326.53
M4	Outside 95% Prediction Interval (%)	9.62%	13.46%	9.62%	9.62%	–
	RMSE	106.92	110.12	146.23	217.26	303.56
	MAE	64.01	66.72	82.51	113.05	326.29
M5	Outside 95% Prediction Interval (%)	9.62%	13.46%	7.69%	9.62%	–
	RMSE	107.70	110.03	145.86	**217.17**	303.55
	MAE	64.54	66.53	82.42	113.73	327.22
M6	Outside 95% Prediction Interval (%)	9.62%	13.46%	11.54%	9.62%	–
	RMSE	106.74	110.14	146.40	217.18	303.52
	MAE	63.87	66.57	82.49	112.70	325.63

**Note**: Bold values identify the lowest RMSE or MAE within each state/Total column across M1-M6. Out-of-sample results are based on 52 rolling one-step-ahead forecasts (MMWR 2024 W09 through 2025 W08).

The percentages of observations outside the 95% prediction intervals were also generally similar across models, ranging from 7.69% to 13.46%. These values were somewhat higher than the expected 5% rate, with the largest percentages occurring in Kansas. Because the out-of-sample assessment consisted of only 52 weekly forecasts, each additional observation outside the prediction interval changes the reported percentage by approximately 1.92 percentage points, which partly explains the identical percentages observed for several models. Given the primary emphasis on out-of-sample predictive accuracy, M3 was selected as the preferred model because it achieved the lowest joint RMSE while also showing strong state-specific performance in Arkansas and Kansas. This result, together with its favorable in-sample performance and the flexibility of the softplus formulation to accommodate both positive and negative covariate effects while maintaining positivity of the conditional centering parameter, supports M3 as the preferred specification for the empirical application.

The 52-week one-step-ahead forecasts from the selected M3 model are displayed in Fig 4 for Arkansas, Kansas, Missouri, and Oklahoma, together with the observed weekly ILI counts and corresponding 95% prediction intervals.

**Fig 4:**
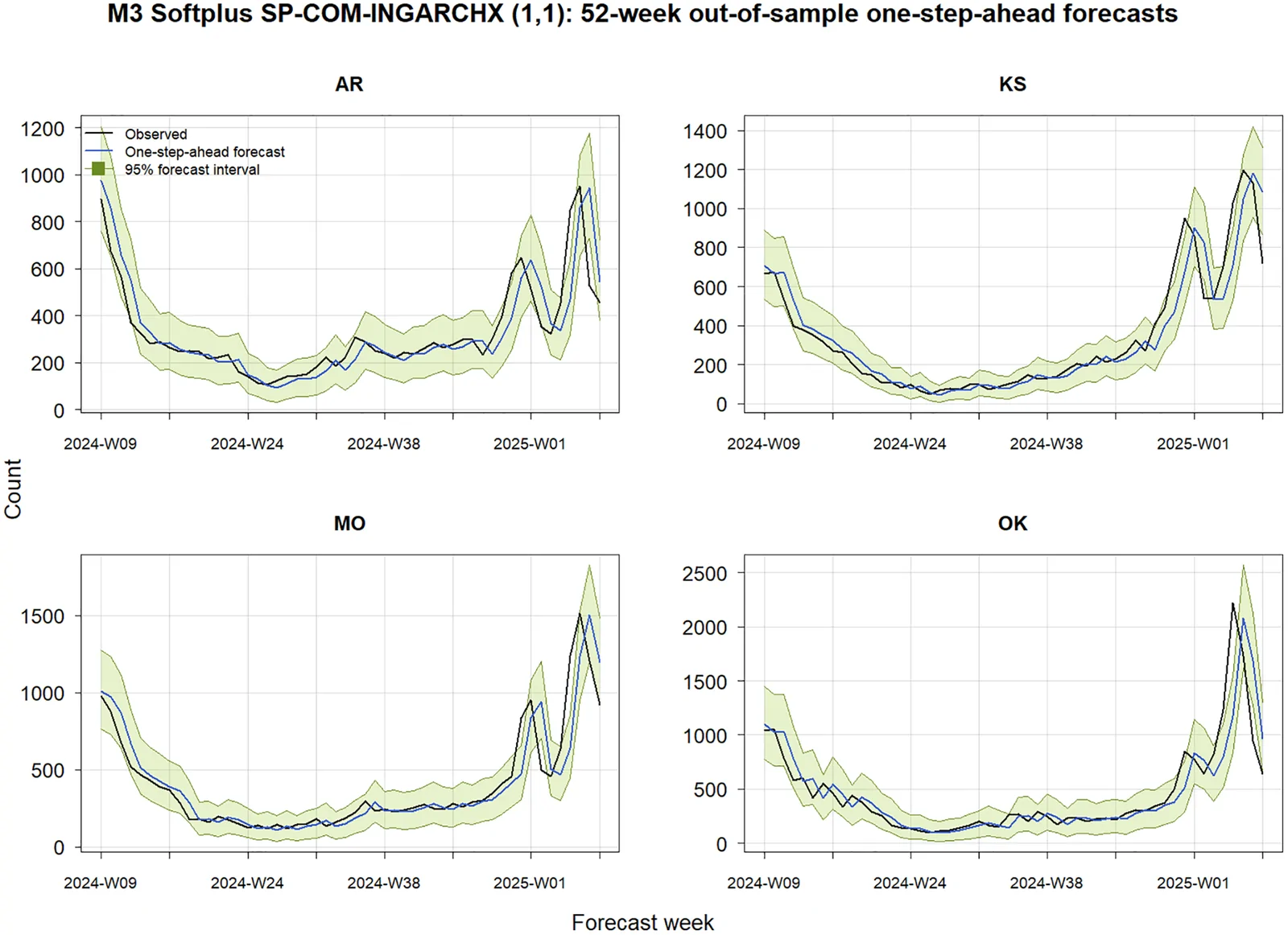
Fifty-two-week out-of-sample one-step-ahead forecasts and 95% prediction intervals from the softplus SP-COM-INGARCHX (1 1) model (M3) for Arkansas, Kansas, Missouri, and Oklahoma.

## 5. Discussion

The primary motivation for this study was to develop a unified spatiotemporal framework for count data that simultaneously accommodates temporal dependence, spatial association, and deviations from the Poisson equidispersion assumption. Such features commonly arise in infectious-disease surveillance, where counts observed in one region may depend on both its previous activity and disease activity in neighboring regions. To address these challenges, we proposed a spatial Conway-Maxwell-Poisson INGARCH (SP-COM-INGARCH) framework in which the conditional distribution follows a COM-Poisson distribution capable of modeling both overdispersion and underdispersion. Three model formulations were considered: the baseline SP-COM-INGARCH model, the identity-link SP-COM-INGARCHX model, and the softplus SP-COM-INGARCHX model. The softplus extension was specifically designed to accommodate covariate effects of either sign while ensuring positivity of the conditional centering parameter.

Model parameters were estimated using conditional maximum likelihood estimation and simulation studies demonstrated generally satisfactory finite-sample performance. Estimation accuracy improved as the time-series length increased, particularly for the INGARCH parameters that govern temporal dependence. Some spatial and dispersion parameters were estimated less precisely under low-count or strongly dispersed conditions. In addition, very large negative covariate effects tended to be estimated closer to zero, although their signs were recovered correctly. These findings suggest that estimates of spatial, dispersion parameters, and large-magnitude covariate-effect should be interpreted cautiously when the available time series is short or contains limited information regarding these components.

The empirical analysis compared the proposed COM-Poisson formulations with analogous spatial generalized-Poisson INGARCH models. Within the COM-Poisson family, incorporating absolute humidity through the identity-link formulation produced only limited improvement over the baseline model. The softplus SP-COM-INGARCHX model, in contrast, achieved the lowest AIC and BIC, the lowest overall in-sample MAE, and prediction-interval coverage close to the nominal level. However, the baseline SP-COM-INGARCH model retained the lowest overall and COVID-period RMSE and the lowest COVID-period MAE. The estimated humidity effects were negative across all four states, demonstrating the practical value of a formulation that accommodates negative covariate effects while preserving positivity. Within the generalized-Poisson family, the baseline SP-GP-INGARCH model was favored by AIC and BIC, while its covariate extensions produced only modest improvements in predictive performance. The softplus formulation therefore provided a clearer empirical benefit within the COM-Poisson family than within the generalized-Poisson family.

All fitted models experienced noticeable difficulty capturing influenza patterns during the COVID-19 period. This likely reflects abrupt changes in disease activity, health-care utilization, population behavior, and public-health interventions that were not fully represented by the spatial, temporal, and humidity components of the fitted models. In the 52-week rolling-origin out-of-sample evaluation, the softplus SP-COM-INGARCHX model achieved the lowest joint RMSE, whereas the baseline SP-COM-INGARCH model achieved the lowest joint MAE. The differences among several models were relatively small and should therefore not be overstated. Nevertheless, when considered together with its likelihood-based fit and ability to incorporate negative humidity effects, the results support the softplus SP-COM-INGARCHX model as the preferred predictive specification. The baseline SP-GP-INGARCH model remains a competitive and more parsimonious alternative. This comparison highlights an important trade-off: the COM-Poisson framework provides greater flexibility for modeling dispersion and covariate effects, whereas the generalized-Poisson framework is computationally simpler.

Overall, the proposed SP-COM-INGARCH framework extends the spatial count time-series literature by integrating temporal dependence, distance-based spatial interaction, flexible dispersion modeling, and covariate effects within a single INGARCH framework. Theoretical results, simulation evidence, and the influenza application collectively demonstrate its usefulness for analyzing and forecasting spatially correlated count processes, particularly when both overdispersion and underdispersion are plausible features of the data.

## 6. Limitations

Despite the strengths of the proposed framework, several limitations should be acknowledged. The empirical application was limited to four neighboring states and used a point-based estimate of absolute humidity from one prespecified location within each state. Consequently, these measurements may not fully represent environmental conditions across each state. Although absolute humidity varied across time and space, its effect was assumed to remain constant over the study period. Other factors, including vaccination, mobility, health-care utilization, public-health interventions, and additional meteorological variables, may account for some of the remaining variation. Future studies could evaluate the framework in larger spatial systems and consider additional covariates, alternative lag specifications, different spatial-weight structures, and time-varying covariate or intervention effects.

Model estimation relies on numerical optimization and may therefore be sensitive to the choice of initial values, particularly because of the model’s dimensionality and dependence structure. Careful convergence diagnostics and, when necessary, multiple starting values are important for obtaining reliable estimates. The present study also focused on the parsimonious first-order specification. Increasing the autoregressive or moving-average orders would introduce additional spatial-count and conditional-parameter lag coefficients, increasing the computational burden and the potential for weak identifiability and convergence difficulties. The empirical performance of such higher-order specifications was not evaluated and remains an area for future research.

Finally, the dispersion parameter was allowed to vary across states but was assumed to remain constant over time. Because influenza exhibits strong seasonality, its dispersion may change across seasons or epidemic periods, potentially shifting between overdispersion and underdispersion. A time-invariant dispersion parameter may not fully capture these changes. Future extensions could therefore incorporate time-varying dispersion and examine comparable softplus covariate formulations for other count distributions.

## 7. Conclusions

This study proposed a flexible framework for modeling spatiotemporal count data by combining a conditional Conway–Maxwell–Poisson distribution with a spatial INGARCH structure. Three formulations were developed, and their parameters were estimated using conditional maximum likelihood. The simulation study demonstrated generally satisfactory finite-sample estimation accuracy, with performance typically improving as the time series length increased, although some spatial and dispersion parameters required more cautious interpretation under challenging conditions. The empirical analysis of weekly influenza-like illnesses counts across four neighboring states further demonstrated the practical applicability of the proposed framework. In particular, the softplus SP-COM-INGARCHX model accommodated negative covariate effects while preserving positivity and achieved the lowest joint out-of-sample RMSE. Although differences in forecasting performance among several models were small, the baseline SP-COM-INGARCH model achieved the lowest joint MAE, and the generalized Poisson models remained competitive as computationally simpler alternatives. Overall, the softplus SP-COM-INGARCHX model provided the most favorable balance of model fit, forecasting performance, and covariate flexibility. These findings support the proposed framework as a useful and interpretable approach for jointly capturing temporal dependence, spatial association, covariate effects, and flexible dispersion in count time series data.

### Use of artificial intelligence tools

During manuscript preparation and revision, the authors used ChatGPT (OpenAI) to assist with language editing, grammatical refinement, organization of text, and improvement of clarity and readability. Artificial intelligence was not used for the study design, statistical methodology, model development, data analysis, or interpretation of results. All AI-assisted text was critically reviewed and verified by the authors, who take full responsibility for the accuracy, integrity, and final content of the submitted work.

## Supporting information

S1 Appendix Proof of stationarity and ergodicity for the SP-COM-INGARCH (1,1) model. (DOCX)

S2 Appendix Simulation Results for the Identity-Link SP-COM-INGARCHX (1,1) Model. (DOCX)
