# Supplementary material for: Modeling spatially dependent count time series data using COM-poisson INGARCH Models
Source: PLoS One. 2026 Oct 6;21(10):e0350497. doi: 10.1371/journal.pone.0350497 (PMC13641397; doi:10.1371/journal.pone.0350497)
Supplement: Supplementary file 1 [file pone.0350497.s001.docx]

# S1 Appendix. Proof of stationarity and ergodicity for the SP-COM-INGARCH (1,1) model

This appendix establishes the stationarity and ergodicity result stated in Section 2.1.4. The proof uses a backward-iteration argument for stochastic recurrence equations, following the general approach of Bougerol [1], adapted to the SP-COM-INGARCH setting.

Let

$$\mathbf{Y}_{t}=\left( Y_{1,t},\ldots,Y_{K,t} \right)^{\mathsf{T}}$$

and

$$\boldsymbol{\mu}_{t}=\left( \mu_{1,t},\ldots,\mu_{K,t} \right)^{\mathsf{T}}.$$

The SP-COM-INGARCH(1,1) recursion can be written as

$$\boldsymbol{\mu}_{t}=\boldsymbol{\omega}+\mathbf{AG}\mathbf{Y}_{t-1}+\mathbf{B}\boldsymbol{\mu}_{t-1},$$

(S1)

where

$$\boldsymbol{\omega}=\left( \omega_{1},\ldots,\omega_{K} \right)^{\mathsf{T}},$$

$$\mathbf{A}=diag\left( \alpha_{1},\ldots,\alpha_{K} \right), \mathbf{B}=diag\left( \beta_{1},\ldots,\beta_{K} \right),$$

and $\mathbf{G}$ is the $K\times K$ spatial weight matrix with entries

$$G_{ij}=\gamma_{ji}.$$

Here, j indexes the source unit and i indexes the receiving unit. The normalization in Equation (12) includes j = i, so the diagonal weight γᵢᵢ is retained. It gives

$$\sum_{j=1}^{K} \gamma_{ji}=1$$

for each $i$. Therefore,

$$\sum_{j=1}^{K} G_{ij}=\sum_{j=1}^{K} \gamma_{ji}=1,$$

so $\mathbf{G}$ is row-stochastic. All entries of $\mathbf{A}$, $\mathbf{B}$, and $\mathbf{G}$ are nonnegative.

Vectors are compared componentwise; that is, $\mathbf{x}\leq\mathbf{y}$ means $x_{i}\leq y_{i}$ for every $i$. The notation $\left| \mathbf{x} \right|$ denotes the vector of componentwise absolute values. Thus, for any nonnegative matrix $\mathbf{C}$,

$$\left| \mathbf{Cx} \right|\leq\mathbf{C}\left| \mathbf{x} \right|.$$

Whenever a scalar norm is used below, $\left\| \left. \mathbf{x} \right\| \right.$denotes any fixed norm on $\mathbb{R}^{K}$.

For spatial unit $i$, let

$$m_{i}\left( u \right)=E\left( Y_{i,t}\mid\mu_{i,t}=u,\nu_{i} \right)$$

denote the exact conditional COM-Poisson mean.

## S1.1 The COM-Poisson mean function

### Lemma S1

Fix $\nu>0$, and suppose that $Y$ follows the reparametrized COM-Poisson distribution

$$P\left( Y=y \right)=\frac{1}{S\left( \mu,\nu\right)}\left( \frac{\mu^{y}}{y!} \right)^{\nu}, y=0,1,2,\ldots,$$

where μ>0 and the normalizing constant is

$$S\left( \mu,\nu\right)=\sum_{r=0}^{\infty} \left( \frac{\mu^{r}}{r!} \right)^{\nu}.$$

Define

$$m\left( \mu\right)=E\left( Y \right)$$

and the variance function

$$V\left( \mu\right)=Var\left( Y \right),$$

Then $m$ is continuously differentiable on $\left( 0,\infty\right)$, and

$$m'\left( \mu\right)=\frac{\nu}{\mu}V\left( \mu\right).$$

(S2)

In particular, $m$ is nondecreasing.

### Proof

Set

$$\theta=\nu\log\mu.$$

The probability mass function can be written as the natural exponential family

$$p\left( y;\theta\right)=exp\left\{ \theta y-\nu\log\left( y! \right)-\kappa\left( \theta\right) \right\},$$

where

$$\kappa\left( \theta\right)=log\left[ \sum_{y=0}^{\infty} \exp\left\{ \theta y-\nu\log\left( y! \right) \right\} \right].$$

For every $\theta\mathbb{\in R}$, the defining series is finite when $\nu>0$. Standard exponential-family identities therefore give

$$\kappa'\left( \theta\right)=E\left( Y \right)$$

and

$$\kappa''\left( \theta\right)=Var\left( Y \right).$$

Since

$$\frac{d\theta}{d\mu}=\frac{\nu}{\mu}$$

the chain rule yields

$$\begin{matrix} m'\left( \mu\right)=\frac{dm}{d\theta}\frac{d\theta}{d\mu} \\ =\kappa''\left( \theta\right)\frac{\nu}{\mu} \\ =\frac{\nu}{\mu}V\left( \mu\right). \end{matrix}$$

Because $V\left( \mu\right)\geq0$, it follows that $m'\left( \mu\right)\geq0$, and hence $m$ is nondecreasing.

For the contraction result, assume that $m_{i}'$ is bounded on the admissible state space $[\omega_{i},\infty)$, and define

$$L_{i}:=\sup_{u\geq\omega_{i}}m_{i}'\left( u \right)<\infty, i=1,\ldots,K.$$

(S3)

Because Lemma S1 shows that $m_{i}'\left( u \right)\geq0$, this is equivalent to defining $L_{i}$ using $\left| m_{i}'\left( u \right) \right|$. Let

$$\mathbf{D}_{L}=diag\left( L_{1},\ldots,L_{K} \right).$$

## S1.2 Monotone coupling

### Lemma S2

Fix $\nu>0$. For $\mu,\mu'>0$, let $Y\left( \mu\right)$ and $Y\left( \mu' \right)$ have reparametrized COM-Poisson distributions with parameters $\left( \mu,\nu\right)$ and $\left( \mu',\nu\right)$, respectively. They can be constructed using a common uniform random variable such that

$$E\left[ \left| Y\left( \mu\right)-Y\left( \mu' \right) \right| \right]=\left| m\left( \mu\right)-m\left( \mu' \right) \right|.$$

(S4)

Consequently, if $\mu,\mu'\geq\omega_{0}$ and

$$L=\sup_{u\geq\omega_{0}}m'\left( u \right)<\infty,$$

then

$$E\left[ \left| Y\left( \mu\right)-Y\left( \mu' \right) \right| \right]\leq L\left| \mu-\mu' \right|.$$

(S5)

### Proof

For $y\geq1$,

$$\frac{p\left( y;\mu\right)}{p\left( y-1;\mu\right)}=\left( \frac{\mu}{y} \right)^{\nu}.$$

Thus, when $\mu'>\mu$,

$$\frac{p\left( y;\mu' \right)/p\left( y;\mu\right)}{p\left( y-1;\mu' \right)/p\left( y-1;\mu\right)}=\left( \frac{\mu'}{\mu} \right)^{\nu}>1.$$

Therefore, the likelihood ratio $p\left( y;\mu' \right)/p\left( y;\mu\right)$ is increasing in $y$. The family has the monotone likelihood-ratio property in $\mu$, and hence

$$Y\left( \mu\right)\leq_{\mathrm{st}}Y\left( \mu' \right)$$

whenever $\mu\leq\mu'$, where $\leq_{\mathrm{st}}$ denotes the usual stochastic order; see Shaked and Shanthikumar [2].

Equivalently,

$$F\left( y;\mu\right)\geq F\left( y;\mu' \right)$$

for every $y$, or

$$P\{Y\left( \mu\right)>y\}\leq P\{Y\left( \mu' \right)>y\}.$$

Let

$$Q\left( u;\mu\right)=inf\left\{ y\in\mathbb{N}_{0}:F\left( y;\mu\right)\geq u \right\}$$

denote the quantile function of the COM-Poisson distribution with parameters $\left( \mu,\nu\right)$. For

$$U\sim Uniform\left( 0,1 \right),$$

define

$$\tilde{Y}\left( \mu\right)=Q\left( U;\mu\right), \tilde{Y}\left( \mu' \right)=Q\left( U;\mu' \right).$$

Stochastic monotonicity implies

$$\tilde{Y}\left( \mu\right)\leq\tilde{Y}\left( \mu' \right) \text{almost surely}$$

whenever $\mu\leq\mu'$. Therefore,

$$\begin{matrix} E\left[ \left| \tilde{Y}\left( \mu\right)-\tilde{Y}\left( \mu' \right) \right| \right]=E\left[ \tilde{Y}\left( \mu' \right)-\tilde{Y}\left( \mu\right) \right] \\ =m\left( \mu' \right)-m\left( \mu\right) \\ =\left| m\left( \mu' \right)-m\left( \mu\right) \right|. \end{matrix}$$

This proves (S4).

By the mean-value theorem, there exists a value $\xi$ between $\mu$ and $\mu'$ such that

$$m\left( \mu\right)-m\left( \mu' \right)=m'\left( \xi\right)\left( \mu-\mu' \right).$$

Hence,

$$\left| m\left( \mu\right)-m\left( \mu' \right) \right|=\left| m'\left( \xi\right) \right| \left| \mu-\mu' \right|\leq L\left| \mu-\mu' \right|.$$

Combining this inequality with (S4) gives

$$E\left[ \left| Y\left( \mu\right)-Y\left( \mu' \right) \right| \right]\leq L\left| \mu-\mu' \right|,$$

which proves (S5).

## S1.3 One-step contraction

Consider two versions of the recursion,

$$\{\boldsymbol{\mu}_{t},\mathbf{Y}_{t}\} \text{and} \{\boldsymbol{\mu}_{t}',\mathbf{Y}_{t}'\},$$

generated using the common-uniform coupling of Lemma S2 at each time and spatial unit.

### Lemma S3

For every $t$,

$$E\left[ \left| \boldsymbol{\mu}_{t}-\boldsymbol{\mu}_{t}' \right| \right]\leq\mathbf{M}E\left[ \left| \boldsymbol{\mu}_{t-1}-\boldsymbol{\mu}_{t-1}' \right| \right],$$

(S6)

where

$$\mathbf{M}=\mathbf{AG}\mathbf{D}_{L}+\mathbf{B}.$$

(S7)

The inequality is componentwise. More generally, for any integer $n\geq1$,

$$E\left[ \left| \boldsymbol{\mu}_{t}-\boldsymbol{\mu}_{t}' \right| \right]\leq\mathbf{M}^{n}E\left[ \left| \boldsymbol{\mu}_{t-n}-\boldsymbol{\mu}_{t-n}' \right| \right].$$

(S8)

### Proof

The two recursions are

$$\boldsymbol{\mu}_{t}=\boldsymbol{\omega}+\mathbf{AG}\mathbf{Y}_{t-1}+\mathbf{B}\boldsymbol{\mu}_{t-1}$$

and

$$\boldsymbol{\mu}_{t}'=\boldsymbol{\omega}+\mathbf{AG}\mathbf{Y}_{t-1}'+\mathbf{B}\boldsymbol{\mu}_{t-1}'.$$

Subtracting the second equation from the first gives

$$\begin{matrix} \boldsymbol{\mu}_{t}-\boldsymbol{\mu}_{t}'=\left( \boldsymbol{\omega}+\mathbf{AG}\mathbf{Y}_{t-1}+\mathbf{B}\boldsymbol{\mu}_{t-1} \right) \\ -\left( \boldsymbol{\omega}+\mathbf{AG}\mathbf{Y}_{t-1}'+\mathbf{B}\boldsymbol{\mu}_{t-1}' \right) \\ =\mathbf{AG}\left( \mathbf{Y}_{t-1}-\mathbf{Y}_{t-1}' \right)+\mathbf{B}\left( \boldsymbol{\mu}_{t-1}-\boldsymbol{\mu}_{t-1}' \right), \end{matrix}$$

because the common intercept $\boldsymbol{\omega}$ cancels.

Taking componentwise absolute values and applying the triangle inequality gives

$$\begin{matrix} \left| \boldsymbol{\mu}_{t}-\boldsymbol{\mu}_{t}' \right|\leq\left| \mathbf{AG}\left( \mathbf{Y}_{t-1}-\mathbf{Y}_{t-1}' \right) \right| \\ +\left| \mathbf{B}\left( \boldsymbol{\mu}_{t-1}-\boldsymbol{\mu}_{t-1}' \right) \right|. \end{matrix}$$

Since $\mathbf{A}$, $\mathbf{G}$, and $\mathbf{B}$ have nonnegative entries,

$$\left| \mathbf{Cx} \right|\leq\mathbf{C}\left| \mathbf{x} \right|$$

for any nonnegative matrix $\mathbf{C}$. Therefore,

$$\left| \boldsymbol{\mu}_{t}-\boldsymbol{\mu}_{t}' \right|\leq\mathbf{AG}\left| \mathbf{Y}_{t-1}-\mathbf{Y}_{t-1}' \right|+\mathbf{B}\left| \boldsymbol{\mu}_{t-1}-\boldsymbol{\mu}_{t-1}' \right|.$$

Taking conditional expectations given $\left( \boldsymbol{\mu}_{t-1},\boldsymbol{\mu}_{t-1}' \right)$ gives

$$\begin{matrix} E\left[ \left| \boldsymbol{\mu}_{t}-\boldsymbol{\mu}_{t}' \right|\mid\boldsymbol{\mu}_{t-1},\boldsymbol{\mu}_{t-1}' \right] \\ \leq\mathbf{AG}E\left[ \left| \mathbf{Y}_{t-1}-\mathbf{Y}_{t-1}' \right|\mid\boldsymbol{\mu}_{t-1},\boldsymbol{\mu}_{t-1}' \right] \\ +\mathbf{B}\left| \boldsymbol{\mu}_{t-1}-\boldsymbol{\mu}_{t-1}' \right|. \end{matrix}$$

By Lemma S2, applied separately to each spatial unit,

$$E\left[ \left| \mathbf{Y}_{t-1}-\mathbf{Y}_{t-1}' \right|\mid\boldsymbol{\mu}_{t-1},\boldsymbol{\mu}_{t-1}' \right]\leq\mathbf{D}_{L}\left| \boldsymbol{\mu}_{t-1}-\boldsymbol{\mu}_{t-1}' \right|.$$

Substituting this bound gives

$$\begin{matrix} E\left[ \left| \boldsymbol{\mu}_{t}-\boldsymbol{\mu}_{t}' \right|\mid\boldsymbol{\mu}_{t-1},\boldsymbol{\mu}_{t-1}' \right] \\ \leq\left( \mathbf{AG}\mathbf{D}_{L}+\mathbf{B} \right)\left| \boldsymbol{\mu}_{t-1}-\boldsymbol{\mu}_{t-1}' \right|. \end{matrix}$$

Applying the law of total expectation yields

$$E\left[ \left| \boldsymbol{\mu}_{t}-\boldsymbol{\mu}_{t}' \right| \right]\leq\left( \mathbf{AG}\mathbf{D}_{L}+\mathbf{B} \right)E\left[ \left| \boldsymbol{\mu}_{t-1}-\boldsymbol{\mu}_{t-1}' \right| \right].$$

With

$$\mathbf{M}=\mathbf{AG}\mathbf{D}_{L}+\mathbf{B},$$

this proves (S6). Iterating the inequality $n$ times gives (S8).

## S1.4 Existence, uniqueness, stationarity, and ergodicity

### Theorem S1

Assume that ωᵢ > 0, αᵢ, βᵢ ≥ 0, and νᵢ > 0 for i = 1,…,K; G is a nonnegative row-stochastic matrix; and the derivative bounds Lᵢ defined in (S3) are finite. Suppose further that

$$\rho\left( \mathbf{AG}\mathbf{D}_{L}+\mathbf{B} \right)<1,$$

(S9)

where $\rho\left( \cdot\right)$ denotes the spectral radius. Then the SP-COM-INGARCH(1,1) recursion admits a unique causal strictly stationary solution with finite first moments. The joint process

$$\left\{ \left( \mathbf{Y}_{t},\boldsymbol{\mu}_{t} \right) \right\}_{t\mathbb{\in Z}}$$

is ergodic.

### Proof

Let

$$\{\mathbf{U}_{t}{\}}_{t\mathbb{\in Z}}$$

be an i.i.d. sequence of $K$-dimensional random vectors with independent $\mathrm{Uniform}\left( 0,1 \right)$ components.

Let

$$\mathbf{x}=\left( x_{1},\ldots,x_{K} \right)^{\mathsf{T}}$$

denote a generic candidate value of the previous intensity vector. For $\mathbf{x}$ satisfying $x_{i}\geq\omega_{i}$ for every $i$, define

$$\mathbf{R}\left( \mathbf{x},\mathbf{U}_{t} \right)=\left( Q_{1}\left( U_{1,t};x_{1},\nu_{1} \right),\ldots,Q_{K}\left( U_{K,t};x_{K},\nu_{K} \right) \right)^{\mathsf{T}}.$$

For a given state $\mathbf{x}$, the map $\mathbf{R}\left( \mathbf{x},\mathbf{U}_{t} \right)$ generates the corresponding vector of COM-Poisson counts through the componentwise quantile representation.

Define

$$\boldsymbol{\Psi}_{t}\left( \mathbf{x} \right)=\boldsymbol{\omega}+\mathbf{AGR}\left( \mathbf{x},\mathbf{U}_{t-1} \right)+\mathbf{Bx}.$$

(S10)

The mapping $\boldsymbol{\Psi}_{t}$ represents one stochastic update of the model: it takes a previous intensity vector $\mathbf{x}$, generates the associated count vector through $\mathbf{R}\left( \mathbf{x},\mathbf{U}_{t-1} \right)$, and returns the next intensity vector.

Indeed, if

$$\mathbf{x}=\boldsymbol{\mu}_{t-1},$$

then

$$\mathbf{R}\left( \boldsymbol{\mu}_{t-1},\mathbf{U}_{t-1} \right)=\mathbf{Y}_{t-1},$$

and therefore

$$\boldsymbol{\Psi}_{t}\left( \boldsymbol{\mu}_{t-1} \right)=\boldsymbol{\mu}_{t}.$$

For fixed $t$ and $n\geq1$, define the backward approximation

$$\boldsymbol{\mu}_{t}^{\left( n \right)}=\boldsymbol{\Psi}_{t}\circ\boldsymbol{\Psi}_{t-1}\circ\cdots\circ\boldsymbol{\Psi}_{t-n+1}\left( \boldsymbol{\omega} \right),$$

(S11)

with

$$\boldsymbol{\mu}_{t}^{\left( 0 \right)}=\boldsymbol{\omega}.$$

The quantity $\boldsymbol{\mu}_{t}^{\left( n \right)}$ is the value obtained at time $t$ when the recursion is initialized at $\boldsymbol{\omega}$ at time $t-n$ and propagated forward using the innovations $\mathbf{U}_{t-n},\ldots,\mathbf{U}_{t-1}$.

The approximations $\boldsymbol{\mu}_{t}^{\left( n+1 \right)}$ and $\boldsymbol{\mu}_{t}^{\left( n \right)}$ differ only in their states at time $t-n$. From that time onward, they are propagated by the same final $n$ random maps. Their states at time $t-n$ are

$$\boldsymbol{\Psi}_{t-n}\left( \boldsymbol{\omega} \right) \text{and} \boldsymbol{\omega},$$

respectively. Lemma S3 therefore gives

$$\begin{matrix} E\left[ \left| \boldsymbol{\mu}_{t}^{\left( n+1 \right)}-\boldsymbol{\mu}_{t}^{\left( n \right)} \right| \right] \\ \leq\mathbf{M}^{n}E\left[ \left| \boldsymbol{\Psi}_{t-n}\left( \boldsymbol{\omega} \right)-\boldsymbol{\omega} \right| \right]. \end{matrix}$$

Because $\{\mathbf{U}_{t}\}$ is i.i.d., the random maps $\{\boldsymbol{\Psi}_{t}\}$ are identically distributed. Hence, the expectation on the right-hand side is the same for every $t$ and $n$. Define

$$\mathbf{c}:=E\left[ \left| \boldsymbol{\Psi}_{0}\left( \boldsymbol{\omega} \right)-\boldsymbol{\omega} \right| \right].$$

Thus,

$$E\left[ \left| \boldsymbol{\mu}_{t}^{\left( n+1 \right)}-\boldsymbol{\mu}_{t}^{\left( n \right)} \right| \right]\leq\mathbf{M}^{n}\mathbf{c}.$$

(S12)

The vector $\mathbf{c}$ measures the expected componentwise displacement produced by one random update from the initial state $\boldsymbol{\omega}$.

Moreover,

$$\boldsymbol{\Psi}_{0}\left( \boldsymbol{\omega} \right)-\boldsymbol{\omega}=\mathbf{AGR}\left( \boldsymbol{\omega},\mathbf{U}_{-1} \right)+\boldsymbol{B\omega}.$$

Each component of $\mathbf{R}\left( \boldsymbol{\omega},\mathbf{U}_{-1} \right)$ has finite expectation $m_{i}\left( \omega_{i} \right)$. The COM-Poisson distribution has finite moments for finite positive parameters; see Zhu [3]. Therefore,

$$\mathbf{c}<\infty.$$

Since

$$\rho\left( \mathbf{M} \right)<1,$$

the matrix series

$$\sum_{n=0}^{\infty} \mathbf{M}^{n}$$

converges. Hence, by (S12),

$$\sum_{n=0}^{\infty} E\left[ \left| \boldsymbol{\mu}_{t}^{\left( n+1 \right)}-\boldsymbol{\mu}_{t}^{\left( n \right)} \right| \right]<\infty$$

componentwise.

Tonelli’s theorem permits the expectation and the nonnegative infinite sum to be interchanged. Therefore,

$$\sum_{n=0}^{\infty} \left| \boldsymbol{\mu}_{t}^{\left( n+1 \right)}-\boldsymbol{\mu}_{t}^{\left( n \right)} \right|<\infty\text{almost surely}.$$

Thus, the sequence $\{\boldsymbol{\mu}_{t}^{\left( n \right)}\}$ is Cauchy and converges almost surely and in $L^{1}$ to a finite random vector, denoted by $\boldsymbol{\mu}_{t}$:

$$\boldsymbol{\mu}_{t}^{\left( n \right)}\to\boldsymbol{\mu}_{t}.$$

Set

$$\mathbf{Y}_{t}^{\left( n \right)}=\mathbf{R}\left( \boldsymbol{\mu}_{t}^{\left( n \right)},\mathbf{U}_{t} \right)$$

and

$$\mathbf{Y}_{t}=\mathbf{R}\left( \boldsymbol{\mu}_{t},\mathbf{U}_{t} \right).$$

Both $\boldsymbol{\mu}_{t}^{\left( n \right)}$ and $\boldsymbol{\mu}_{t}$ depend only on

$$\mathbf{U}_{t-1},\mathbf{U}_{t-2},\ldots$$

and are therefore independent of $\mathbf{U}_{t}$. By Lemma S2,

$$E\left[ \left| \mathbf{Y}_{t}^{\left( n \right)}-\mathbf{Y}_{t} \right| \right]\leq\mathbf{D}_{L}E\left[ \left| \boldsymbol{\mu}_{t}^{\left( n \right)}-\boldsymbol{\mu}_{t} \right| \right]\to\mathbf{0}.$$

Therefore,

$$\mathbf{Y}_{t}^{\left( n \right)}\to\mathbf{Y}_{t} \text{in }L^{1}.$$

For every $n\geq1$,

$$\boldsymbol{\mu}_{t}^{\left( n \right)}=\boldsymbol{\omega}+\mathbf{AG}\mathbf{Y}_{t-1}^{\left( n-1 \right)}+\mathbf{B}\boldsymbol{\mu}_{t-1}^{\left( n-1 \right)}.$$

Passing to the $L^{1}$ limit gives

$$\boldsymbol{\mu}_{t}=\boldsymbol{\omega}+\mathbf{AG}\mathbf{Y}_{t-1}+\mathbf{B}\boldsymbol{\mu}_{t-1}.$$

Moreover,

$$\mathbf{Y}_{t}=\mathbf{R}\left( \boldsymbol{\mu}_{t},\mathbf{U}_{t} \right),$$

and $\mathbf{U}_{t}$ is independent of the past. It follows that, conditionally on the past, the components of $\mathbf{Y}_{t}$ are independent and have the required COM-Poisson distributions with parameters $\left( \mu_{i,t},\nu_{i} \right)$.

The construction also shows that $\boldsymbol{\mu}_{t}$ is a measurable function of

$$\mathbf{U}_{t-1},\mathbf{U}_{t-2},\ldots,$$

so the solution is causal. Since all coefficients and counts are nonnegative,

$$\boldsymbol{\mu}_{t}\geq\boldsymbol{\omega} \text{almost surely}.$$

The backward construction is shift invariant. Consequently, there exists a measurable function $H$ such that

$$\left( \mathbf{Y}_{t},\boldsymbol{\mu}_{t} \right)=H\left( \mathbf{U}_{t},\mathbf{U}_{t-1},\mathbf{U}_{t-2},\ldots\right)$$

for every $t$. The function $H$ summarizes the entire backward construction and maps the current and past innovation sequence into the corresponding pair $\left( \mathbf{Y}_{t},\boldsymbol{\mu}_{t} \right)$.

Thus, the joint process is a measurable Bernoulli shift of an i.i.d. sequence. It is therefore strictly stationary and ergodic; see Krengel [4] and Straumann and Mikosch [5].

To establish uniqueness, let

$$\left\{ \left( \mathbf{Y}_{t}^{*},\boldsymbol{\mu}_{t}^{*} \right) \right\}_{t\mathbb{\in Z}}$$

be another causal stationary solution satisfying

$$E\left[ \parallel\boldsymbol{\mu}_{0}^{*}\parallel\right]<\infty,$$

realized using the same innovation sequence. By Lemma S3, for any $n\geq1$,

$$E\left[ \left| \boldsymbol{\mu}_{t}-\boldsymbol{\mu}_{t}^{*} \right| \right]\leq\mathbf{M}^{n}E\left[ \left| \boldsymbol{\mu}_{t-n}-\boldsymbol{\mu}_{t-n}^{*} \right| \right].$$

Stationarity and the finite first-moment assumption imply

$$E\left[ \left| \boldsymbol{\mu}_{t-n}-\boldsymbol{\mu}_{t-n}^{*} \right| \right]\leq E\left[ \left| \boldsymbol{\mu}_{0} \right| \right]+E\left[ \left| \boldsymbol{\mu}_{0}^{*} \right| \right]<\infty.$$

Because

$$\mathbf{M}^{n}\to\mathbf{0}$$

as $n\to\infty$, it follows that

$$E\left[ \left| \boldsymbol{\mu}_{t}-\boldsymbol{\mu}_{t}^{*} \right| \right]=\mathbf{0}.$$

Therefore,

$$\boldsymbol{\mu}_{t}=\boldsymbol{\mu}_{t}^{*} \text{almost surely}.$$

The common-uniform representation then gives

$$\mathbf{Y}_{t}=\mathbf{Y}_{t}^{*} \text{almost surely}.$$

Finally, the backward construction yields

$$E\left[ \parallel\boldsymbol{\mu}_{t}\parallel\right]\leq\parallel\boldsymbol{\omega}\parallel+\sum_{n=0}^{\infty} E\left[ \left\| \boldsymbol{\mu}_{t}^{\left( n+1 \right)}-\boldsymbol{\mu}_{t}^{\left( n \right)} \right\| \right]<\infty.$$

For each $i$, Lemma S1 and the definition of $L_{i}$ give

$$m_{i}\left( \mu_{i,t} \right)\leq m_{i}\left( \omega_{i} \right)+L_{i}\left( \mu_{i,t}-\omega_{i} \right).$$

Because

$$E\left( Y_{i,t} \right)=E\left[ m_{i}\left( \mu_{i,t} \right) \right],$$

we obtain

$$E\left( Y_{i,t} \right)\leq m_{i}\left( \omega_{i} \right)+L_{i}E\left( \mu_{i,t}-\omega_{i} \right)<\infty.$$

Hence,

$$E\left[ \parallel\mathbf{Y}_{t}\parallel\right]<\infty,$$

completing the proof.

## S1.5 Verifiable sufficient conditions

### Corollary S1

Recall from (S7) that

$$\mathbf{M}=\mathbf{AG}\mathbf{D}_{L}+\mathbf{B}.$$

If either the row-wise conditions in (S13) or the simpler conditions in (S14) hold, then ρ(M) < 1 and the conclusions of Theorem S1 follow.

### Proof

Because $\mathbf{M}$ is nonnegative,

$$\rho\left( \mathbf{M} \right)\leq\parallel\mathbf{M}\parallel_{\infty}=\max_{1\leq i\leq K}\left\{ \alpha_{i}\sum_{j=1}^{K} \gamma_{ji}L_{j}+\beta_{i} \right\}.$$

Therefore, the row-wise conditions

$$\alpha_{i}\sum_{j=1}^{K} \gamma_{ji}L_{j}+\beta_{i}<1, i=1,\ldots,K,$$

(S13)

are sufficient for the spectral contraction condition.

Let

$$L_{\max}=\max_{1\leq j\leq K}L_{j}.$$

Because

$$\sum_{j=1}^{K} \gamma_{ji}=1,$$

we have

$$\sum_{j=1}^{K} \gamma_{ji}L_{j}\leq L_{\max}.$$

Thus,

$$L_{\max}\alpha_{i}+\beta_{i}<1, i=1,\ldots,K,$$

(S14)

is a simpler, although potentially more restrictive, sufficient condition. This completes the proof.

When $\nu_{i}=1$ for every $i$, the conditional distribution is Poisson,

$$m_{i}\left( u \right)=u,$$

and

$$L_{i}=1.$$

The spectral condition becomes

$$\rho\left( \mathbf{AG}+\mathbf{B} \right)<1,$$

while the directly verifiable row-wise condition reduces to

$$\alpha_{i}+\beta_{i}<1, i=1,\ldots,K.$$

This agrees with the familiar sufficient stability condition for the univariate mean-parameterized Poisson INGARCH model [6]. The conditions above are sufficient rather than necessary; they impose geometric contraction in $L^{1}$, which is stronger than strict stationarity alone.

## References

1. Bougerol, P. (1993). Kalman filtering with random coefficients and contractions. *SIAM Journal on Control and Optimization*, *31*(4), 942-959.
2. Shaked, M., & Shanthikumar, J. G. (Eds.). (2007). *Stochastic orders*. New York, NY: Springer New York.
3. Zhu, F. (2012). Modeling time series of counts with COM-Poisson INGARCH models. *Mathematical and Computer Modelling*, *56*(9-10), 191-203.
4. Krengel, U. (2011). *Ergodic theorems* (Vol. 6). Walter de Gruyter.
5. Straumann D, Mikosch T. Quasi-maximum-likelihood estimation in conditionally heteroscedastic time series: a stochastic recurrence equations approach. The Annals of Statistics. 2006;34(5):2449–2495. <https://doi.org/10.1214/009053606000000803>
6. Fokianos, K., Rahbek, A., & Tjøstheim, D. (2009). Poisson autoregression. *Journal of the American Statistical Association*, *104*(488), 1430-1439.
