# Supplementary material for: Modeling spatially dependent count time series data using COM-poisson INGARCH Models
Source: PLoS One. 2026 Oct 6;21(10):e0350497. doi: 10.1371/journal.pone.0350497 (PMC13641397; doi:10.1371/journal.pone.0350497)
Supplement: Supplementary file 2 [file pone.0350497.s002.docx]

**S2 Appendix. Simulation Results for the Identity-Link SP-COM-INGARCHX (1,1) Model**

This appendix presents the detailed finite-sample simulation results, based on 1000 Monte Carlo replications, for the identity-link SP-COM-INGARCHX (1,1) model under three overdispersion scenarios (O1–O3) and three underdispersion scenarios (U1–U3). For time-series lengths of (N=500) and (N=1000), Tables S1–S6 report the true parameter values, mean estimates, biases, and mean absolute errors (MAEs).

**Table S1:** SP-COM-INGARCHX (1,1) Simulation Results Under Overdispersion: Scenario O1

| **Parameter** | **Truth** | **Time Series Length N = 500** | | | **Time Series Length N = 1000** | | |
| --- | --- | --- | --- | --- | --- | --- | --- |
|  |  | **Mean Estimate** | **Bias** | **MAE** | **Mean Estimate** | **Bias** | **MAE** |
| $\omega_{1}$ | 1.0000 | 1.1616 | 0.1616 | 0.2625 | 1.1741 | 0.1741 | 0.2311 |
| $\omega_{2}$ | 1.3000 | 1.4743 | 0.1743 | 0.3370 | 1.4808 | 0.1808 | 0.2845 |
| $\omega_{3}$ | 1.1000 | 1.3774 | 0.2774 | 0.3445 | 1.3841 | 0.2841 | 0.3128 |
| $\omega_{4}$ | 1.5000 | 1.5987 | 0.0987 | 0.3303 | 1.6207 | 0.1207 | 0.2578 |
| $\alpha_{1}$ | 0.1100 | 0.1078 | -0.0022 | 0.0399 | 0.1078 | -0.0022 | 0.0292 |
| $\alpha_{2}$ | 0.0900 | 0.0863 | -0.0037 | 0.0401 | 0.0872 | -0.0028 | 0.0288 |
| $\alpha_{3}$ | 0.1000 | 0.0992 | -0.0008 | 0.0396 | 0.1001 | 0.0001 | 0.0288 |
| $\alpha_{4}$ | 0.0800 | 0.0784 | -0.0016 | 0.0362 | 0.0789 | -0.0011 | 0.0272 |
| $\beta_{1}$ | 0.1900 | 0.1905 | 0.0005 | 0.1039 | 0.1926 | 0.0026 | 0.0836 |
| $\beta_{2}$ | 0.2100 | 0.2189 | 0.0089 | 0.1177 | 0.2187 | 0.0087 | 0.0942 |
| $\beta_{3}$ | 0.1800 | 0.1864 | 0.0064 | 0.0987 | 0.1908 | 0.0108 | 0.0746 |
| $\beta_{4}$ | 0.2200 | 0.2164 | -0.0036 | 0.1234 | 0.2147 | -0.0053 | 0.0902 |
| $\tau$ | 0.7500 | 0.8203 | 0.0703 | 0.3014 | 0.8217 | 0.0717 | 0.2260 |
| $\kappa$ | 0.3000 | 0.3476 | 0.0476 | 0.1208 | 0.3432 | 0.0432 | 0.1071 |
| $\nu_{1}$ | 0.6500 | 0.8903 | 0.2403 | 0.2403 | 0.8867 | 0.2367 | 0.2367 |
| $\nu_{2}$ | 0.5500 | 0.7265 | 0.1765 | 0.1765 | 0.7185 | 0.1685 | 0.1685 |
| $\nu_{3}$ | 0.4500 | 0.6515 | 0.2015 | 0.2015 | 0.6517 | 0.2017 | 0.2017 |
| $\nu_{4}$ | 0.7000 | 0.8504 | 0.1504 | 0.1504 | 0.8444 | 0.1444 | 0.1444 |
| $\phi_{1}$ | 0.3000 | 0.3275 | 0.0275 | 0.1714 | 0.3102 | 0.0102 | 0.1254 |
| $\phi_{2}$ | 0.3500 | 0.3706 | 0.0206 | 0.2057 | 0.3615 | 0.0115 | 0.1498 |
| $\phi_{3}$ | 0.2500 | 0.2907 | 0.0407 | 0.1676 | 0.2794 | 0.0294 | 0.1343 |
| $\phi_{4}$ | 0.4000 | 0.4388 | 0.0388 | 0.2022 | 0.4099 | 0.0099 | 0.1370 |

**Table S2:** SP-COM-INGARCHX (1,1) Simulation Results Under Overdispersion: Scenario O2

| **Parameter** | **Truth** | **Time Series Length N = 500** | | | **Time Series Length N = 1000** | | |
| --- | --- | --- | --- | --- | --- | --- | --- |
|  |  | **Mean Estimate** | **Bias** | **MAE** | **Mean Estimate** | **Bias** | **MAE** |
| $\omega_{1}$ | 10.0000 | 10.0367 | 0.0367 | 1.3099 | 10.0697 | 0.0697 | 0.9053 |
| $\omega_{2}$ | 15.0000 | 15.1545 | 0.1545 | 1.7841 | 15.0307 | 0.0307 | 1.2956 |
| $\omega_{3}$ | 12.0000 | 12.2590 | 0.2590 | 1.4739 | 12.0066 | 0.0066 | 1.0315 |
| $\omega_{4}$ | 17.0000 | 17.0413 | 0.0413 | 1.9297 | 17.1703 | 0.1703 | 1.4544 |
| $\alpha_{1}$ | 0.2900 | 0.2845 | -0.0055 | 0.0348 | 0.2867 | -0.0033 | 0.0250 |
| $\alpha_{2}$ | 0.3400 | 0.3347 | -0.0053 | 0.0374 | 0.3370 | -0.0030 | 0.0262 |
| $\alpha_{3}$ | 0.2700 | 0.2632 | -0.0068 | 0.0352 | 0.2697 | -0.0003 | 0.0249 |
| $\alpha_{4}$ | 0.3100 | 0.3047 | -0.0053 | 0.0332 | 0.3079 | -0.0021 | 0.0255 |
| $\beta_{1}$ | 0.3100 | 0.3076 | -0.0024 | 0.0496 | 0.3076 | -0.0024 | 0.0333 |
| $\beta_{2}$ | 0.2600 | 0.2561 | -0.0039 | 0.0480 | 0.2586 | -0.0014 | 0.0337 |
| $\beta_{3}$ | 0.3300 | 0.3222 | -0.0078 | 0.0484 | 0.3249 | -0.0051 | 0.0340 |
| $\beta_{4}$ | 0.2900 | 0.2902 | 0.0002 | 0.0491 | 0.2854 | -0.0046 | 0.0348 |
| $\tau$ | 1.5000 | 1.5174 | 0.0174 | 0.0526 | 1.5158 | 0.0158 | 0.0462 |
| $\kappa$ | 0.5000 | 0.5033 | 0.0033 | 0.0636 | 0.5051 | 0.0051 | 0.0465 |
| $\nu_{1}$ | 0.6000 | 0.6126 | 0.0126 | 0.0329 | 0.6081 | 0.0081 | 0.0229 |
| $\nu_{2}$ | 0.5000 | 0.5079 | 0.0079 | 0.0271 | 0.5061 | 0.0061 | 0.0197 |
| $\nu_{3}$ | 0.4500 | 0.4597 | 0.0097 | 0.0246 | 0.4558 | 0.0058 | 0.0173 |
| $\nu_{4}$ | 0.6000 | 0.6093 | 0.0093 | 0.0317 | 0.6058 | 0.0058 | 0.0222 |
| $\phi_{1}$ | 2.5000 | 2.6265 | 0.1265 | 0.5013 | 2.5248 | 0.0248 | 0.3468 |
| $\phi_{2}$ | 3.0000 | 3.1340 | 0.1340 | 0.6434 | 3.1028 | 0.1028 | 0.4816 |
| $\phi_{3}$ | 2.0000 | 2.2163 | 0.2163 | 0.4593 | 2.1856 | 0.1856 | 0.3646 |
| $\phi_{4}$ | 3.5000 | 3.5965 | 0.0965 | 0.7314 | 3.5673 | 0.0673 | 0.5076 |

**Table S3:** SP-COM-INGARCHX (1,1) Simulation Results Under Overdispersion: Scenario O3

| **Parameter** | **Truth** | **Time Series Length N = 500** | | | **Time Series Length N = 1000** | | |
| --- | --- | --- | --- | --- | --- | --- | --- |
|  |  | **Mean Estimate** | **Bias** | **MAE** | **Mean Estimate** | **Bias** | **MAE** |
| $\omega_{1}$ | 1000.0000 | 986.4860 | -13.5140 | 22.4228 | 987.3621 | -12.6379 | 23.0671 |
| $\omega_{2}$ | 1500.0000 | 1492.0000 | -8.0000 | 25.4470 | 1496.2592 | -3.7408 | 25.4870 |
| $\omega_{3}$ | 1200.0000 | 1193.6078 | -6.3922 | 22.8319 | 1194.2477 | -5.7523 | 24.0948 |
| $\omega_{4}$ | 1700.0000 | 1695.1573 | -4.8427 | 25.9839 | 1698.7091 | -1.2909 | 26.3112 |
| $\alpha_{1}$ | 0.2800 | 0.2798 | -0.0002 | 0.0046 | 0.2804 | 0.0004 | 0.0051 |
| $\alpha_{2}$ | 0.3000 | 0.2995 | -0.0005 | 0.0042 | 0.2994 | -0.0006 | 0.0050 |
| $\alpha_{3}$ | 0.3200 | 0.3196 | -0.0004 | 0.0049 | 0.3197 | -0.0003 | 0.0058 |
| $\alpha_{4}$ | 0.2900 | 0.2900 | 0.0000 | 0.0045 | 0.2895 | -0.0005 | 0.0049 |
| $\beta_{1}$ | 0.3200 | 0.3174 | -0.0026 | 0.0073 | 0.3167 | -0.0033 | 0.0079 |
| $\beta_{2}$ | 0.3000 | 0.2975 | -0.0025 | 0.0072 | 0.2974 | -0.0026 | 0.0078 |
| $\beta_{3}$ | 0.2800 | 0.2762 | -0.0038 | 0.0074 | 0.2759 | -0.0041 | 0.0073 |
| $\beta_{4}$ | 0.3100 | 0.3067 | -0.0033 | 0.0075 | 0.3080 | -0.0020 | 0.0065 |
| $\tau$ | 1.2000 | 1.2098 | 0.0098 | 0.0351 | 1.2033 | 0.0033 | 0.0264 |
| $\kappa$ | 0.8000 | 0.8064 | 0.0064 | 0.0147 | 0.8043 | 0.0043 | 0.0128 |
| $\nu_{1}$ | 0.7500 | 0.7476 | -0.0024 | 0.0173 | 0.7463 | -0.0037 | 0.0172 |
| $\nu_{2}$ | 0.7000 | 0.6954 | -0.0046 | 0.0200 | 0.6952 | -0.0048 | 0.0197 |
| $\nu_{3}$ | 0.6000 | 0.5883 | -0.0117 | 0.0243 | 0.5910 | -0.0090 | 0.0224 |
| $\nu_{4}$ | 0.7000 | 0.6911 | -0.0089 | 0.0223 | 0.6904 | -0.0096 | 0.0240 |
| $\phi_{1}$ | 200.0000 | 234.4873 | 34.4873 | 41.3036 | 236.9564 | 36.9564 | 45.7784 |
| $\phi_{2}$ | 300.0000 | 334.3171 | 34.3171 | 48.0989 | 335.8698 | 35.8698 | 50.1245 |
| $\phi_{3}$ | 240.0000 | 280.1060 | 40.1060 | 45.5177 | 286.1785 | 46.1785 | 52.0084 |
| $\phi_{4}$ | 340.0000 | 372.6292 | 32.6292 | 45.5243 | 363.4744 | 23.4744 | 34.1841 |

**Table S4:** SP-COM-INGARCHX (1,1) Simulation Results Under Underdispersion: Scenario U1

| **Parameter** | **Truth** | **Time Series Length N = 500** | | | **Time Series Length N = 1000** | | |
| --- | --- | --- | --- | --- | --- | --- | --- |
|  |  | **Mean Estimate** | **Bias** | **MAE** | **Mean Estimate** | **Bias** | **MAE** |
| $\omega_{1}$ | 1.0000 | 1.0360 | 0.0360 | 0.1420 | 1.0304 | 0.0304 | 0.1004 |
| $\omega_{2}$ | 1.4000 | 1.4110 | 0.0110 | 0.1911 | 1.4138 | 0.0138 | 0.1235 |
| $\omega_{3}$ | 1.2000 | 1.2014 | 0.0014 | 0.1750 | 1.2076 | 0.0076 | 0.1200 |
| $\omega_{4}$ | 1.6000 | 1.6029 | 0.0029 | 0.1757 | 1.6226 | 0.0226 | 0.1307 |
| $\alpha_{1}$ | 0.1200 | 0.1189 | -0.0011 | 0.0386 | 0.1211 | 0.0011 | 0.0279 |
| $\alpha_{2}$ | 0.1000 | 0.0958 | -0.0042 | 0.0379 | 0.0972 | -0.0028 | 0.0259 |
| $\alpha_{3}$ | 0.0800 | 0.0819 | 0.0019 | 0.0344 | 0.0783 | -0.0017 | 0.0240 |
| $\alpha_{4}$ | 0.1100 | 0.1084 | -0.0016 | 0.0364 | 0.1092 | -0.0008 | 0.0284 |
| $\beta_{1}$ | 0.1800 | 0.1775 | -0.0025 | 0.0698 | 0.1817 | 0.0017 | 0.0488 |
| $\beta_{2}$ | 0.2000 | 0.2005 | 0.0005 | 0.0751 | 0.1994 | -0.0006 | 0.0485 |
| $\beta_{3}$ | 0.2200 | 0.2178 | -0.0022 | 0.0835 | 0.2195 | -0.0005 | 0.0549 |
| $\beta_{4}$ | 0.1700 | 0.1737 | 0.0037 | 0.0605 | 0.1677 | -0.0023 | 0.0402 |
| $\tau$ | 0.7500 | 0.9096 | 0.1596 | 0.3247 | 0.9080 | 0.1580 | 0.2747 |
| $\kappa$ | 0.3000 | 0.3339 | 0.0339 | 0.0893 | 0.3292 | 0.0292 | 0.0734 |
| $\nu_{1}$ | 1.3000 | 1.5828 | 0.2828 | 0.2830 | 1.5670 | 0.2670 | 0.2670 |
| $\nu_{2}$ | 1.5000 | 1.6819 | 0.1819 | 0.1855 | 1.6674 | 0.1674 | 0.1684 |
| $\nu_{3}$ | 1.7000 | 1.9402 | 0.2402 | 0.2428 | 1.9277 | 0.2277 | 0.2281 |
| $\nu_{4}$ | 1.4000 | 1.5505 | 0.1505 | 0.1571 | 1.5448 | 0.1448 | 0.1469 |
| $\phi_{1}$ | 0.3000 | 0.3115 | 0.0115 | 0.1166 | 0.3031 | 0.0031 | 0.0888 |
| $\phi_{2}$ | 0.3500 | 0.3629 | 0.0129 | 0.1327 | 0.3611 | 0.0111 | 0.0936 |
| $\phi_{3}$ | 0.2500 | 0.2574 | 0.0074 | 0.1075 | 0.2552 | 0.0052 | 0.0774 |
| $\phi_{4}$ | 0.4000 | 0.4096 | 0.0096 | 0.1526 | 0.4010 | 0.0010 | 0.1070 |

**Table S5:** SP-COM-INGARCHX (1,1) Simulation Results Under Underdispersion: Scenario U2

| **Parameter** | **Truth** | **Time Series Length N = 500** | | | **Time Series Length N = 1000** | | |
| --- | --- | --- | --- | --- | --- | --- | --- |
|  |  | **Mean Estimate** | **Bias** | **MAE** | **Mean Estimate** | **Bias** | **MAE** |
| $\omega_{1}$ | 10.0000 | 9.9958 | -0.0042 | 0.8327 | 10.0377 | 0.0377 | 0.6031 |
| $\omega_{2}$ | 15.0000 | 15.0854 | 0.0854 | 1.1914 | 15.0847 | 0.0847 | 0.8469 |
| $\omega_{3}$ | 12.0000 | 11.9726 | -0.0274 | 0.9169 | 12.0237 | 0.0237 | 0.6582 |
| $\omega_{4}$ | 17.0000 | 17.2375 | 0.2375 | 1.3721 | 17.1215 | 0.1215 | 0.9784 |
| $\alpha_{1}$ | 0.3200 | 0.3165 | -0.0035 | 0.0278 | 0.3174 | -0.0026 | 0.0205 |
| $\alpha_{2}$ | 0.2800 | 0.2761 | -0.0039 | 0.0273 | 0.2781 | -0.0019 | 0.0196 |
| $\alpha_{3}$ | 0.3500 | 0.3466 | -0.0034 | 0.0266 | 0.3484 | -0.0016 | 0.0200 |
| $\alpha_{4}$ | 0.3000 | 0.2948 | -0.0052 | 0.0276 | 0.2969 | -0.0031 | 0.0201 |
| $\beta_{1}$ | 0.2800 | 0.2779 | -0.0021 | 0.0263 | 0.2779 | -0.0021 | 0.0189 |
| $\beta_{2}$ | 0.3300 | 0.3283 | -0.0017 | 0.0283 | 0.3277 | -0.0023 | 0.0191 |
| $\beta_{3}$ | 0.2500 | 0.2474 | -0.0026 | 0.0230 | 0.2464 | -0.0036 | 0.0165 |
| $\beta_{4}$ | 0.3100 | 0.3062 | -0.0038 | 0.0286 | 0.3080 | -0.0020 | 0.0195 |
| $\tau$ | 1.5000 | 1.5103 | 0.0103 | 0.0433 | 1.5085 | 0.0085 | 0.0388 |
| $\kappa$ | 0.5000 | 0.5087 | 0.0087 | 0.0449 | 0.5048 | 0.0048 | 0.0316 |
| $\nu_{1}$ | 1.4000 | 1.4216 | 0.0216 | 0.0550 | 1.4079 | 0.0079 | 0.0377 |
| $\nu_{2}$ | 1.6000 | 1.6174 | 0.0174 | 0.0726 | 1.6091 | 0.0091 | 0.0486 |
| $\nu_{3}$ | 1.8000 | 1.8296 | 0.0296 | 0.0870 | 1.8137 | 0.0137 | 0.0604 |
| $\nu_{4}$ | 1.5000 | 1.5119 | 0.0119 | 0.0620 | 1.5080 | 0.0080 | 0.0420 |
| $\phi_{1}$ | 2.5000 | 2.5447 | 0.0447 | 0.3183 | 2.5586 | 0.0586 | 0.2576 |
| $\phi_{2}$ | 3.0000 | 3.0351 | 0.0351 | 0.3748 | 3.0651 | 0.0651 | 0.3026 |
| $\phi_{3}$ | 2.0000 | 2.1295 | 0.1295 | 0.3020 | 2.1483 | 0.1483 | 0.2741 |
| $\phi_{4}$ | 3.5000 | 3.5928 | 0.0928 | 0.4490 | 3.5912 | 0.0912 | 0.3302 |

**Table S6:** SP-COM-INGARCHX (1,1) Simulation Results Under Underdispersion: Scenario U3

| **Parameter** | **Truth** | **Time Series Length N = 500** | | | **Time Series Length N = 1000** | | |
| --- | --- | --- | --- | --- | --- | --- | --- |
|  |  | **Mean Estimate** | **Bias** | **MAE** | **Mean Estimate** | **Bias** | **MAE** |
| $\omega_{1}$ | 1000.0000 | 987.1283 | -12.8717 | 20.9901 | 992.4724 | -7.5276 | 27.9695 |
| $\omega_{2}$ | 1500.0000 | 1492.5838 | -7.4162 | 22.6523 | 1484.5445 | -15.4555 | 23.0359 |
| $\omega_{3}$ | 1200.0000 | 1191.6608 | -8.3392 | 20.8424 | 1197.1369 | -2.8631 | 17.8954 |
| $\omega_{4}$ | 1700.0000 | 1691.0349 | -8.9651 | 26.3222 | 1675.6049 | -24.3951 | 37.3671 |
| $\alpha_{1}$ | 0.2700 | 0.2698 | -0.0002 | 0.0042 | 0.2684 | -0.0016 | 0.0043 |
| $\alpha_{2}$ | 0.3100 | 0.3093 | -0.0007 | 0.0043 | 0.3093 | -0.0007 | 0.0039 |
| $\alpha_{3}$ | 0.2900 | 0.2903 | 0.0003 | 0.0045 | 0.2886 | -0.0014 | 0.0048 |
| $\alpha_{4}$ | 0.3300 | 0.3298 | -0.0002 | 0.0044 | 0.3304 | 0.0004 | 0.0048 |
| $\beta_{1}$ | 0.3300 | 0.3273 | -0.0027 | 0.0074 | 0.3309 | 0.0009 | 0.0069 |
| $\beta_{2}$ | 0.2900 | 0.2881 | -0.0019 | 0.0071 | 0.2880 | -0.0020 | 0.0075 |
| $\beta_{3}$ | 0.3200 | 0.3157 | -0.0043 | 0.0079 | 0.3175 | -0.0025 | 0.0082 |
| $\beta_{4}$ | 0.2700 | 0.2673 | -0.0027 | 0.0066 | 0.2684 | -0.0016 | 0.0071 |
| $\tau$ | 1.2000 | 1.2074 | 0.0074 | 0.0344 | 1.2213 | 0.0213 | 0.0444 |
| $\kappa$ | 0.8000 | 0.8061 | 0.0061 | 0.0134 | 0.8075 | 0.0075 | 0.0169 |
| $\nu_{1}$ | 1.2000 | 1.1989 | -0.0011 | 0.0216 | 1.1970 | -0.0030 | 0.0279 |
| $\nu_{2}$ | 1.2500 | 1.2504 | 0.0004 | 0.0257 | 1.2389 | -0.0111 | 0.0250 |
| $\nu_{3}$ | 1.3000 | 1.2944 | -0.0056 | 0.0300 | 1.2930 | -0.0070 | 0.0242 |
| $\nu_{4}$ | 1.2000 | 1.1984 | -0.0016 | 0.0242 | 1.1978 | -0.0022 | 0.0202 |
| $\phi_{1}$ | 200.0000 | 234.5007 | 34.5007 | 41.5606 | 217.5045 | 17.5045 | 31.1007 |
| $\phi_{2}$ | 300.0000 | 329.5824 | 29.5824 | 44.6704 | 351.5795 | 51.5795 | 64.0499 |
| $\phi_{3}$ | 240.0000 | 277.3857 | 37.3857 | 43.4118 | 282.4864 | 42.4864 | 49.9290 |
| $\phi_{4}$ | 340.0000 | 374.6586 | 34.6586 | 45.7240 | 384.1500 | 44.1500 | 52.3808 |
